# An Optimal Flow Admission and Routing Control Policy for Resource Constrained Networks

**DOI:** 10.3390/s20226566

**Published:** 2020-11-17

**Authors:** Essia Hamouda

**Affiliations:** J. H. Brown College of Business & Public Administration, California State University, San Bernardino, CA 92407, USA; ehamouda@csusb.edu

**Keywords:** wireless communication, performance optimization, markov decision process, energy efficiency, threshold routing, sensor networks

## Abstract

Overloaded network devices are becoming an increasing problem especially in resource limited networks with the continuous and rapid increase of wireless devices and the huge volume of data generated. Admission and routing control policy at a network device can be used to balance the goals of maximizing throughput and ensuring sufficient resources for high priority flows. In this paper we formulate the admission and routing control problem of two types of flows where one has a higher priority than the other as a Markov decision problem. We characterize the optimal admission and routing policy, and show that it is a state-dependent threshold type policy. Furthermore, we conduct extensive numerical experiments to gain more insight into the behavior of the optimal policy under different systems’ parameters. While dynamic programming can be used to solve such problems, the large size of the state space makes it untractable and too resource intensive to run on wireless devices. Therefore, we propose a fast heuristic that exploits the structure of the optimal policy. We empirically show that the heuristic performs very well with an average reward deviation of 1.4% from the optimal while being orders of magnitude faster than the optimal policy. We further generalize the heuristic for the general case of a system with *n* (n>2) types of flows.

## 1. Introduction

Efficient resource utilization is a primary problem in resource constrained networks. In wireless sensor networks (WSNs) for instance the issue of energy efficiency is crucial to ensure network connectivity and quality of service. In WSNs, sensor nodes are generally deployed to transmit sensitive information in a timely manner. They rely on neighboring nodes to relay traffic to a given destination while operating on limited battery capacity. Energy is used when a node is listening, receiving, or transmitting. If a node’s battery is depleted, neighboring nodes become incapable of relaying and transmitting urgent traffic through the node. More importantly, if said node belongs to the optimal path, a less efficient path will need to be computed which reduces network throughput and consumes more of the scares resources. It is expected that some sensor networks will be deployed over large and inhospitable areas [1,2,3,4,5,6]. Since these networks may not be accessible following deployment, it is crucial that implemented admission and routing policies are resource efficient (i.e., consume minimal energy).

**Figure 1:**
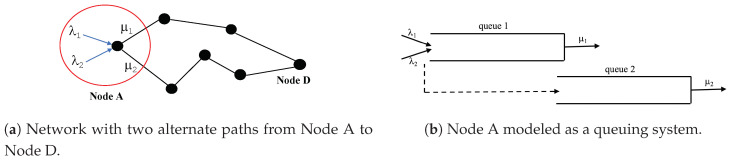
The queueing system at node *A* where type-i,i∈{1,2} arrivals join queue 1. Instead of being aborted from the system, packets are routed to queue 2; $\mu_{2}\leq \mu_{1}$. Served at queue 1 or at queue 2, packets will reach the same destination D over alternate paths.

Consider a sensor node *A* with two available paths to the same destination as shown in Figure 1. We define a task as the transmission of a single flow from a relaying node to the final destination. We say that a task is successful if its corresponding flow is treated to the full extent - that is, all packets that belong to the same flow reach their final destination. Consider the scenario where sensor node *A* is tasked with relaying two flows. To maximize task success, it may be more efficient to treat one task to a full extent and reject or treat the second task partially than to partially treat both tasks. For instance, suppose a node is sending information about two events to a control center (CC) simultaneously: an attack on a battlefield and a fire in another nearby region. The CC would prefer to receive full information about one flow and act on it, rather than receive partial information about both flows that would be discarded. Hence, sometimes rejecting a flow may be perceived as more beneficial than accepting a flow and partially transmitting it due to lack of resources. This saves energy consumption while transmitting a flow to the full extent.

In this paper, we model node *A* (Figure 1) as a queueing system where accepted packets belong to two different flows. We assume that one of the flows has higher priority than the other. Once a packet of a given flow is accepted to the system, it joins queue 1 and it is guaranteed service to the full extent independent of its type. Instead of being rejected or preempted from service, packets have the option to be served at a slower server behind a second queue (queue 2). Hence, the packet is transmitted over a less rewarding path. Transmitted type-*i* packets are rewarded $r_{i},\;i\in \left\{1,2\right\},\;r_{1}>r_{2}>0$ if served at queue 1, and $r_{3}\leq r_{2}$ if served at queue 2. Using the less rewarding path not only minimizes the number of rejected packets from the system, but also maximizes the chance that both flows will be treated to the full extent. Most importantly, allowing packets to be served at queue 2, allows the extension of the life of the path behind queue 1 (the efficient path).

We are interested in finding ways by which node *A* can, through its local decision policy, accept or reject and decide which path to choose to transmit packets. The objective is to minimize energy consumption and maximize the number of flows served, hence maximizing network throughput.

Such decision control mechanism is fundamental to a variety of other interesting applications. For example, consider the case where a node experiences a flood of traffic as a sign of it being compromised. A node can be subject to a SYN flood where an attacker attempts to fill the backlog queue of a victim machine’s Transmission Control Protocol server (TCP) [7,8]. This results in resource depletion that renders the node unresponsive to legitimate traffic. By recognizing such flood of traffic, a node may either classify it as high priority traffic to identify the attacker and take the appropriate measures, or as low priority traffic and route it to another queue with a slower server.

This problem finds applications not only in computers and communication networks but in various other fields as well. Blockchain-based applications for instance, suffer from high computational and storage expenses, negatively impacting overall performance and scalability [9]. Therefore, work has been done to move computation and data off the chain (Off-chain). Off-chain transactions (i.e., high priority traffic) can be executed instantly and usually have low or no transaction fee. However, on-chain transactions (i.e., low priority traffic) can have a lengthy lag time depending on the network load and on the number of transactions waiting in the queue to be confirmed. Similarly, control of multi-class queueing systems has received significant attention in supply chain management and manufacturing systems ([10,11,12] and references therein). One of the main tools for such control problems is to characterize a performance measure of interest and use optimization methods to find the optimal control policy [13,14,15]. An agorithm for optimal pricing and admission control is proposed in [14,16].

In this paper, we develop a dynamic programming formulation of the Admission and Routing Control (ARC) problem, that maximizes the network throughput by extending the life (the resources) of the efficient path and thus the number of flows serviced to the full extent. We formulate the ARC problem as a Markov decision process (MDP) [17] and characterize the optimal policy under the Poisson traffic model. In particular, we show that the ARC policy that maximizes the expected reward is stationary and is a state-dependent threshold type policy. While dynamic programming can be used to solve such problems, the large size of the state space makes it untractable and too resource intensive to run on network devices and especially on wireless devices. Therefore, we propose a fast heuristic that exploits the structure of the optimal policy. Much of the computation required for our method can be done off-line, and the real-time computation requires no more than a table lookup. Furthermore, computing the parameters of the heuristic control policy is orders of magnitude faster than computing the optimal. We empirically show that the heuristic performs very well with an average reward deviation of 1.4% from the optimal while being orders of magnitude faster than the optimal policy. We further generalize the heuristic for the general case of a system with *n* (*n* > 2) types of flows. We believe our heuristic is general enough to be widely applicable and can be implemented in realtime. Although the method we propose applies to general network resource constraints, we consider energy limitations as our motivating application.

The rest of the paper is organized as follows. In Section 2, we provide a literature review. In Section 3, we describe the model and provide its mathematical formulation. In Section 4, we develop properties of the expected reward value function and characterize the optimal *ARC* policy. In Section 5, we analyze the behavior of the optimal policy through extensive numerical experiments. In Section 6, we propose a heuristic control policy, for the general case of *n* types of packets, and conduct extensive numerical experiments, for the case of 2 types of flows, in order to assess its performance compared to the optimal policy. In Section 7, we summarize our findings and propose future directions of this work.

## 2. Literature

In this section, we review the existing literature pertaining to resource constrained environments in WSNs. This is by no means exhaustive; it is only indicative of the interest and the applications.

Extensive work has been done to address the problem of energy saving with respect to WSNs. In [18,19,20,21], controlled mobility is used to extend the network lifetime. Algorithms for self-organization of sensor networks have been proposed [22,23] to minimize the risk of data loss during transmission and to maximize the battery life of individual sensors. Various coverage optimization protocols have been studied [24,25] where a number of sensor nodes are deployed to ensure adequate coverage of a region. Using a coverage optimization protocol, nodes with overlapping sensing areas are turned off to reduce energy consumption. We refer the readers to the following survey [26] of the various other energy efficient coverage techniques. Research in [19] focused on shortest path algorithms to optimize energy consumption. However, using the shortest path may lead to an increase in the ratio of lost packets [27]. In [28], research focused on scheduling sensor nodes to switch on and off, depending on the queue size, to reduce energy consumption. However, switching nodes from an idle to a busy state and vice versa has been a major portion of the power consumption [29].

Numerous works have also treated admission control policies relevant to WSNs. Several algorithms have been used by Internet routers to decide on packets admission and rejection to manage queues and minimize congestion in TCP [30]. In Tail Drop [31], when the queue reaches its maximum capacity, the newly arriving packets are rejected independent of their types. Though traffic may belong to different flows, it is not differentiated, and each packet is treated identically. When segments are lost, the TCP sender enters slow-start, which reduces throughput in that TCP session. A more severe problem occurs when segments from multiple TCP flows are dropped causing global synchronization - that is, all of the involved TCP senders enter slow-start. This problem can be mitigated by routing segments from one flow (the lower priority flow for instance) to a different queue, rather than discarding one segment from each flow. In Weighted Random Early Detection (WRED) [32], flows are differentiated and treated depending on their type. Packets with a higher IP address precedence are less likely to be dropped than packets with a lower precedence. Thus, higher priority packets are delivered with a higher probability than lower priority packets. As in TCP, there is no guarantee that discarded packets belong to the same flow.

Queuing theory has also been widely used in network optimization [33,34,35]. In [36], a queueing network model was used to analyze and study the performance of a mobile WSN. In [37,38], models implementing admission control mechanisms to manage scarce radio resources in WSNs are described in the form of a queueing system with unreliable devices. Work in [39] proposed an energy saving mechanism that controls the ON/OFF state of a sensor node. A sensor node enters an OFF state (multiple fixed duration vacation periods) as soon as its queue is empty and is turned on (changes to an ON state) only when its queue size reaches a threshold value of packets. A M/M/1-type queueing model with a control mechanism is proposed in [40] as a tool to reduce power consumption in WSNs. The tool switches a sensor node from an OFF mode to an ON mode only when its queue size reaches or exceeds a given size.

In order to achieve quality of service in a multi-class two parallel queue system, work in [41,42,43] dedicates a server to the high priority flow. In a resource constrained environment, such a model is not efficient. The dedicated server may be idle for a long time waiting, and consuming resources, for the high priority traffic to arrive while other servers may be congested. A different approach is considered in research that focuses on a single server and two classes of jobs. To minimize the sum of the holding/processing and switching costs, work in [44] switches serving between two classes of traffic. In [45] the authors consider a two-class single server preemptive priority queue where jobs can be denied admission to the system and can be aborted from service. Aborted jobs can rejoin the queue and resume service at a later time. Service does not have to restart but it continues from the step it was aborted from. In [46], an expulsion/scheduling control mechanism is proposed for a single class M/M/2 queueing system with non-identical servers. It was further extended in  [47] to a multiple class system where job preemption is allowed.

The concept of termination control, studying a single server one-class workload model is introduced in [48]. In this work, the service of a job may be aborted before the job has received full service, and may be removed at any point in time from the queue. The authors further show that optimal threshold (acceptance and termination) policies exist.

A common characteristic of these methods is that they may find applications in areas such as workflow and assembly lines. However, they are not applicable to communication networks. One key element of communication networks is that once a packet transmission starts, it cannot be aborted and resume its service at a later time. A packet can be either fully transmitted or put back in the queue before its service starts. Moreover, in resource constrained networks, once a packet is queued or starts service, processing and computation resources are consumed. Preempting its service (transmission) and returning it to the queue for its service to restart at a later time, only consumes more of the scarce resources.

A recurrent assumption of existing work related to admission control is that low priority packets are denied admission to the queue when higher priority packets are already present. In the event they are accepted, low priority packets can be at any time rejected from the queue, or aborted from service in favor of a higher priority packet [45]. Another option in multiserver systems is to restrict sending the high priority flow over one of the available paths (generally the optimal one). These approaches may work well in certain applications such as delay tolerant networks and networks with unlimited resources. They are also applicable in general applications such as in workflow and in assembly lines. However, in resource constrained computer networks, once a packet is accepted to the system, resource (energy, computation, memory) consumption starts.

## 3. Model Description and Formulation

### 3.1. Model Description

We consider the system given in Figure 1. We model node *A* as a two-class queueing system and assume that type-*i*, i∈{1,2}, packets arrive at node *A* according to independent Poisson process with arrival rate $\lambda_{i}\geq 0$, respectively. We assume that node *A* has two paths that lead to the destination *D*: an energy efficient path, through queue 1, and a less energy efficient path through queue 2. We assume that the service at queue 1 and at queue 2 are exponentially distributed with rates $\mu_{1}$ and $\mu_{2}$ respectively, where $\mu_{1}\geq \mu_{2}$. We assume that type-1 packets have higher priority than type-2 packets hence, they are always accepted to queue 1 upon arrival. However, an admission control mechanism at node *A* decides to accept or reject type-2 packets. Arrivals (type-1 and accepted type-2 packets) join queue 1. At any event, arrival or service completion, the decision maker can decide to route a packet to queue 2 or to serve it at queue 1. This decision is made to give service advantage to the higher priority packets to be served at queue 1 and to be transmitted over the more energy efficient path. In summary, the system is controlled in 3-ways: (1) all type-1 packets are always accepted to the system, (2) an admission policy at node *A* decides to accept or reject a newly arriving type-2 packet, (3) a routing policy at node *A* decides to route type-i,i∈{1,2} packet to queue 2 or to serve it at queue 1. However, type-1 packets are always given service priority at queue 1.

We assume that the decision maker has complete state information, i.e., it knows the instantaneous number of packets of type-i,i∈{1,2} in queue 1 and the total number of packets in queue 2. Thus, the structure of the system is that of a Markovian decision process [49]. As such, we propose to formulate the admission and routing problem as a MDP and use the value iteration technique to characterize the form of the optimal policy. We formulate the problem by defining the states, the transition structure and the feasible actions.

**State.** The state of the system is described by the vector $x=(x_{1},x_{2},x_{3})$ where $x_{i},\;i\in \left\{1,2\right\}$ is the number of type-*i* packets in queue 1 and $x_{3}$ is the number of packets (type-1 and/or type-2) in queue 2.

**Events.** We distinguish three possible events: (1) the arrival of a new packet, (2) the service completion at queue 1, and (3) the service completion at queue 2.

**Decisions.** If the event is an arrival, then if the packet is a type-1, it is automatically accepted to queue 1 and this changes the state $\left(x_{1},x_{2},x_{3}\right)$ into state $\left(x_{1}+1,x_{2},x_{3}\right)$. If alternatively, it is a type-2 packet, then a decision has to be made to accept or reject the newly arrived packet. If the packet is accepted, then this changes the state $\left(x_{1},x_{2},x_{3}\right)$ to $\left(x_{1},x_{2}+1,x_{3}\right)$. If it is rejected, the state does not change. Next, the decision is either to serve a packet in queue 1 or to route it to queue 2. The idea here is, if there are type-1 packets in queue 1, they are given higher service priority at queue 1. Hence type-2 packets will be served at queue 1 only if there are no type-1 packets in queue 1.

One may argue, since there are two queues why not dedicate queue 1 to type-1 packets and queue 2 to type-2 packets? The main reason for not using this approach is so that type-2 packets will not be deprived from using the more efficient path when there are no type-1 packets in queue 1. This also allows type-1 packets to be served at queue 2 in the event queue 1 is heavily congested or when the server at queue 2 is idle.

**Costs and rewards.** Packets earn a reward upon service completion. Packets served at queue 1 receive a type-dependent reward $r_{i},\;i\in \left\{1,2\right\}$. Since type-1 packets have a higher priority than type-2 packets they receive a higher reward $r_{1}\geq r_{2}>0$. Packets served at queue 2 receive a reward $r_{3}$ ($0<r_{3}<r_{2}$) independent of their type.

Packets admitted to the system are also subject to a holding and processing cost *h*, incurred while waiting in the queue or while being served. We assume these costs are linear in the number of packets in the system and are type independent namely, yh≥0 per unit of time when there are $y=\sum_{i=1}^{3}x_{i}$ packets present in the queues. In addition, each time a packet is admitted to the system, type independent admission cost c≥0 is incurred. Rejecting packets is free of charge. Moreover, routing type-2 packets to queue 2 is free of charge while routing type-1 has a positive switching cost $c_{2}$. Imposing a positive switching cost on type-1 packets is intended to discourage these packets from being routed to queue 2, and use the less efficient path especially, when there are type-2 packets in queue 1.

For a reward to be collected and for the model to make sense, the cost incurred by a type-*i* packet served at queue *i* must be smaller than the reward it collects at the queue. When served at queue 1 a packet reward is $r_{i}>c+h/\mu_{1}$. When served at queue 2 a packet reward is $r_{3}>c+h/\mu_{2}+c_{2}I_{\left\{i=1\right\}},\;i\in \left\{1,2\right\}$ where the indicator $I_{\left\{i=1\right\}}=1$ if the packet is type-1 otherwise, it is equal to zero. As our application is related to energy optimization in sensor networks, we assume that all the costs and rewards are in units of energy. The cost *h* can be interpreted as the energy consumed to process and maintain a packet in the queue. The cost *c* is the energy consumed to receive a packet and $c_{2}$ is the energy consumed to switch or move a packet from queue 1 to queue 2. Rewards can be interpreted as the energy saved by successfully transmitting a packet compared to rejecting it.

**Criterion.** The objective is to maximize the expected discounted reward resulting from accepting, routing and servicing flows to completion over an infinite horizon.

**Uniformization.** In order to convert the continuous problem into a discrete one, we follow [50]’s uniformization technique. We adjust the transition rates of the embedded Markov chain of the system so that the transition times between decision times is a sequence of independent exponentially distributed random variables with mean $\frac{1}{\beta }$, where $\beta =\alpha +\lambda_{1}+\lambda_{2}+\mu_{1}+\mu_{2}$. Then with probability $\lambda_{i}/\beta >0,i=1,2$, a transition concerns the arrival of a type-*i* packet, with probability $\mu_{j}/\beta >0,\;j=1,2$ concerns a service completion at queue *j* and with probability α/β>0, the process terminates. Without loss of generality, we scale the time line so that the rate β=1.

**Discounting.** We discount future rewards at a rate α≥0, (i.e., rewards at time *t* are multiplied by $e^{\left(\alpha t\right)}$). This is equivalent to a process that lasts an exponentially distributed time with mean 1/α after which, there will be no more arrivals or service completions.

Note that node *A* in Figure 1, can be modeled as a single-shared-queue system. In this case, a routing decision can be made only when a packet reaches the head of the queue, leading to the head-of-the-line blocking (HOL) problem [51]. As such, single shared queue devices are perceived to have low performance due to the HOL blocking [52,53]. This is the main reason why network devices generally use separate queues per output port. In this work, modeling node *A* as a two-queue system mitigates the HOL problem, especially that the routing decision is made not only right before service but also at the arrival of a packet.

### 3.2. Model Formulation

In the following, we summarize and complete the model in terms of a mathematical formulation. Let $w_{n}\left(x\right)$ be the expected discounted reward of responding to an event given that the system has reached state x following *n* state transitions starting from a randomly chosen initial state (i.e., $w_{0}\left(x\right)=0$ for all x≥0, where 0 is the zero vector of dimension 3 and the inequality x≥0 is taken component-wise).

   **Admission**: Let $T_{a_{i}}w_{n-1}\left(x\right)$ denote the expected discounted reward when an arrival of type-*i* packet event occurs and the system is in state x. Let $e_{k}$ be the *k-th* unit vector of dimension 3. An arrival of type-2 is accepted to the system only if the difference in reward between accepting the packet and rejecting it is positive i.e., $w_{n}\left(x+e_{2}\right)-c\geq w_{n}\left(x\right)$. Recall that type-1 packets are never rejected. Thus, for x≥0,

$$\begin{array}{ccc} & T_{a_{1}}w_{n-1}\left(x\right) & =w_{n-1}\left(x+e_{1}\right)-c\\ & T_{a_{2}}w_{n-1}\left(x\right) & =max\{w_{n-1}\left(x+e_{2}\right)-c,w_{n-1}\left(x\right)\} \end{array}$$

   **Service:** When the system is in state x, a service decision of a packet at queue 1 is made as follows. If it is a type-1 packet, it proceeds with no delay to service at server 1. If it is type-2 packet, it is served at server 1 only if there are no type-1 packets in queue 1. In queue 2, packets are served on a first-come-first-serve independent of their type. We define the service operators $T_{i}$ at queue i,i∈{1,2} as follows:

$$\begin{array}{cc} & T_{1}w_{n-1}\left(x\right)=\left\{\begin{array}{c} w_{n-1}\left(x\right)\;\mathrm{if}\;\;x_{1}=x_{2}=0\\ w_{n-1}\left(x-e_{1}\right)+r_{1}\;\mathrm{if}\;\;x_{1}>0\\ w_{n-1}\left(x-e_{2}\right)+r_{2}\;\mathrm{if}\;\;x_{1}=0\;\mathrm{and}\;x_{2}>0 \end{array}\right. \end{array}$$

and

$$\begin{array}{cc} & T_{2}w_{n-1}\left(x\right)=\left\{\begin{array}{cc} w_{n-1}\left(x\right) & \;\;\;\mathrm{if}\;\;x_{3}=0\\ w_{n-1}\left(x-e_{3}\right)+r_{3} & \;\;\;\mathrm{if}\;\;x_{3}>0 \end{array}\right. \end{array}$$

Let $T_{s}w_{n-1}\left(x\right)$ denote the expected discounted reward when the current state is x and an arrival or a service completion event occurs. Note that $T_{s}w_{n-1}\left(x\right)$, given by Equation (1), represents the expected reward assuming no routing to queue 2 occurred.

(1) $$\begin{array}{c} T_{s}w_{n-1}\left(x\right)=\sum_{i=1}^{2}\left(\lambda_{i}T_{a_{i}}w_{n-1}\left(x\right)+\mu_{i}T_{i}w_{n-1}\left(x\right)\right)-h\sum_{j=1}^{3}x_{i} \end{array}$$

   **Routing:** A type-1 packet may be routed to queue 2 only if no type-2 packets are in queue 1 ($x_{2}=0$) and it is more rewarding to route the packet to queue 2 than to keep it in queue 1. However, type-2 packets can be routed to queue 2 when $x_{2}>0$ and when it is more rewarding to do so. Let $T_{r}w_{n}\left(x\right)$ denote the expected discounted reward when the current state is x and a routing decision to queue 2 is to occur.

(2) $$\begin{array}{cc} & T_{r}w_{n-1}\left(x\right)=\left\{\begin{array}{cc} max\{w_{n-1}\left(x-e_{1}+e_{3}\right)-c_{2},w_{n-1}\left(x\right)\}\; & \;\;\;\mathrm{if}\;x_{2}=0,\;x_{1}>0\\ max\{w_{n-1}\left(x-e_{2}+e_{3}\right),w_{n-1}\left(x\right)\}\; & \;\;\;\mathrm{if}\;x_{2}>0 \end{array}\right. \end{array}$$

The optimal expected discounted reward at state x is given by Equation (3). It is implied that at any event, arrival or service completion, the decision maker can decide to route a packet to queue 2 or serve it at queue 1.

(3) $$\begin{array}{ccc} w_{n}\left(x\right)= & max(T_{s}w_{n-1}\left(x\right),T_{r}w_{n-1}\left(x\right)) & \;\;\;\;\;\mathrm{for}\;\;x_{1}+x_{2}>0 \end{array}$$

## 4. Characterization of the Optimal Arc Policy

To characterize the optimal policy, we use the value iteration technique introduced in [54,55], by recursively evaluating $w_{n}$ using Equation (3) for n≥0. We prove by induction that if some structural properties of the discounted reward function $w_{n}$ are satisfied, then these properties are also satisfied for $w_{n+1}$ and therefore, they hold for all n≥0. As *n* tends to infinity, the optimal policy converges to the unique optimal policy. This convergence result is ensured by Theorem 8.10.1 in [17]. The convergence to the optimal policy is an important result in the MDP literature. It is based on showing that the iteration from $w_{n}$ to $w_{n+1}$ is a contraction mapping as stated in Theorem 6.2.3 in [17]. This Theorem also proves that the optimal infinite horizon policy is independent of the choice of $w_{0}$ and this is why one can simply choose $w_{0}\left(x\right)=0$.

### 4.1. Reward Function Properties

  Solving the optimality Equation (3) analytically is untractable. Hence, in order to characterize the structure of the optimal policy, we show that the optimal reward function satisfies a set of properties which allow us to infer the structure of the optimal policy. The properties are listed and interpreted below.

**Property** **1.** $w_{n}\left(x+e_{i}\right)-w_{n}\left(x\right)\geq w_{n}\left(x+2e_{i}\right)-w_{n}\left(x+e_{i}\right),\;\;\mathrm{for}\;i\in \left\{1,2,3\right\}$.

Property 1 implies that $w_{n}\left(x\right)$ is concave in each of the state variables $x_{i}$. In other words, it implies that the marginal reward (i.e., $w_{n}\left(x+e_{i}\right)-w_{n}\left(x\right)$) of an additional packet of type-i,i∈{1,2} in queue 1 is non-increasing in the number of packets $x_{i}$ for a fixed $x_{j},j\neq i$ and fixed number of packets, $x_{3}$ in queue 2. It also implies that the marginal reward of an additional packet in queue 2 is non-increasing in the number of packets $x_{3}$ for a fixed number of packets of type-i,i∈{1,2} in queue 1.

**Property** **2.** $w_{n}\left(x+e_{i}+e_{j}\right)-w_{n}\left(x+e_{j}\right)\geq w_{n}\left(x+2e_{i}\right)-w_{n}\left(x+e_{i}\right),\;\;\mathrm{for}\;i\neq j\;\mathrm{and}\;i,j\in \left\{1,2,3\right\}$.

Property 2, for i=2 and j=3, states that the marginal reward of an additional type-2 packet in queue 1 is non-increasing in $x_{2}$. Therefore, routing a type-2 packet to queue 2 is less rewarding than servicing it at queue 1. Similarly, Property 2, when i=1 and j=3, states that the marginal reward of an additional type-1 packet in queue 1 is non-increasing in $x_{1}$. Consequently, routing a type-1 packet to queue 2 is less rewarding than servicing it at queue 1. The other cases have similar interpretations.

**Property** **3.** $w_{n}\left(x+e_{j}\right)-w_{n}\left(x\right)\geq w_{n}\left(x+e_{i}+e_{j}\right)-w_{n}\left(x+e_{i}\right),\;\;\mathrm{for}\;i\neq j\;\mathrm{and}\;i,j\in \left\{1,2,3\right\}$.

Property 3 states that the marginal reward of an additional type-*i* packet is non-increasing in $x_{j},\;i,\;j\in \left\{1,2\right\}$ and i≠j for fixed $x_{i}$. Similarly, the marginal value of an additional packet in queue 1 is non-increasing in $x_{j},j\in \left\{1,2\right\}$ for fixed queue 2 size. Mathematically, Property 3 indicates that the reward value function is sub-modular.

### 4.2. Reward Function Bounds

Since all accepted packets are guaranteed to be served at either queue, packets will collect a reward upon service completion as long as $r_{i}>c+h/\mu_{1}$ and $r_{3}>c+h/\mu_{2}+c_{2}I_{\left\{i=1\right\}},i\in \left\{1,2\right\}$. However, since the reward depends on the packet type and on the queue where the packet resides, in this subsection, we bound the reward collected by packet type. We make use of sample path approach [56] to prove the following propositions.

**Proposition** **1.** *For all n≥0 and x≥0, the difference in reward of serving a type-2 packet at queue 1 does not exceed $r_{2}$.*

$$\begin{array}{c} w_{n}\left(x+e_{2}\right)-w_{n}\left(x\right)\leq r_{2},\;x_{1}=0 \end{array}$$

**Proof.**  Using a sample path analysis, let two instances $\Pi_{1}$ and $\Pi_{2}$ of the policy where $\Pi_{1}$ starts at state $x+e_{2}$ and $\Pi_{2}$ starts at state x. $\Pi_{1}$ will follow the actions of the optimal policy and $\Pi_{2}$ will copy the actions of $\Pi_{1}$. An arrival to both instances changes the rewards equally (every arrival is charged a cost of *c*). In the event of a departure from state $x+e_{2}$, due to service completion at queue 1, in this case, we must have $x_{1}=0$, otherwise type-1 takes priority in service, immediately afterwards $\Pi_{2}$ and $\Pi_{1}$ become identical, so a reward of $r_{2}$ is generated. The departure can also be a route to queue 2. In this case, since there is no switching cost for type-2 packets, the reward does not change. □

**Proposition** **2.** *For all n≥0 and x≥0, the difference in reward of serving a packet at queue 2 does not exceed $r_{3}$.*

$$\begin{array}{c} w_{n}\left(x+e_{3}\right)-w_{n}\left(x\right)\leq r_{3} \end{array}$$

**Proof.**  Using a sample path analysis, let two instances $\Pi_{1}$ and $\Pi_{2}$ of the policy where $\Pi_{1}$ starts at state $x+e_{3}$ and $\Pi_{2}$ starts at state x. $\Pi_{1}$ will follow the actions of the optimal policy and $\Pi_{2}$ will copy the actions of $\Pi_{1}$. A departure from both instances changes the rewards equally (every departure is rewarded $r_{3}$ independent of the packet type). Hence, upon a departure from queue 2 at state $x+e_{3}$, $\Pi_{2}$ and $\Pi_{1}$ become identical so the difference in reward is at most $r_{3}$. □

**Proposition** **3.** *For all n≥0 and x≥0, the difference in reward to serve a type-1 packet at queue 1 does not exceed $r_{1}$.*

$$\begin{array}{c} w_{n}\left(x+e_{1}\right)-w_{n}\left(x\right)\leq r_{1} \end{array}$$

**Proof.**  Using a sample path analysis, let two instances of the policy where one ($\Pi_{1}$) starts at state $x+e_{1}$ and the other ($\Pi_{2}$) starts at state x. $\Pi_{1}$ will follow the actions of the optimal policy and $\Pi_{2}$ will copy the actions of $\Pi_{1}$. An arrival to both instances changes the rewards equally (every arrival is charged a cost of *c*). In the event of a departure from state $x+e_{1}$, due to service completion at queue 1 (immediately afterwards $\Pi_{2}$ and $\Pi_{1}$ become identical), a reward of $r_{1}$ is generated (since type-1 takes priority over type-2, the departure will be of type-1 unless $x_{1}=0$). So the difference in reward is at most $r_{1}$. The departure can also be a route to queue 2. Note that a routing in both instances changes the reward equally by the switching cost of $c_{2}<r_{1}$. □

**Proposition** **4.** *For all n≥0 and x≥0, the difference in reward of serving a packet at queue 1 and at queue 2 is larger than $r_{i}-r_{3},i\in \left\{1,2\right\}$*

$$\begin{array}{c} w_{n}\left(x+e_{i}\right)-w_{n}\left(x+e_{3}\right)\geq r_{i}-r_{3},\;\;i\in \left\{0,1\right\} \end{array}$$

**Proof.**  Using a sample path analysis, we first consider the case where i=1 and prove $w_{n}\left(x+e_{1}\right)-w_{n}\left(x+e_{3}\right)\geq r_{1}-r_{3}$. Let two instances $\Pi_{1}$ and $\Pi_{2}$ of the policy where $\Pi_{1}$ starts at state $x+e_{3}$ and instance $\Pi_{2}$ starts at state $x+e_{1}$. Instance $\Pi_{1}$ will follow the optimal policy and instance $\Pi_{2}$ will copy the actions of $\Pi_{1}$. That is, if $\Pi_{1}$ routes its packet, then $\Pi_{2}$ routes its packets, and if $\Pi_{1}$ takes its packet into service, then $\Pi_{2}$ takes its packet into service. For both Instances, while packets are still in the system, their costs and rewards are the same. Hence, the difference in reward between the two instances is zero. However, if a packet is served, a reward of $r_{3}$ in $\Pi_{1}$ is collected and a reward of $r_{1}$ in $\Pi_{2}$ is collected. Hence, the difference in reward between the two instances is $r_{1}-r_{3}>0$.

The proof of the case where i=2, is very similar to the above proof. It suffices to replace $e_{1}$ by $e_{2}$ and $r_{1}$ by $r_{2}$. □

Note that in this work, the admission cost is not as relevant as the reward as it is packet type-independent. However, the problem can be easily generalized to assigning type-dependent costs $c_{a_{i}}>0,\;i\in \left\{1,2\right\},\;(c_{a_{1}}\neq c_{a_{2}}$). On the other hand, the switching cost $c_{2}$ is important for type-1 packets as they are charged only in the event they are routed to queue 2.

We conclude this section with the main results of the paper as illustrated in the following Theorem.

**Theorem** **1.** *There exists a stationary optimal policy for any initial state $x=(x_{1},x_{2},x_{3})$ such that:*

- ***Admission policy*** *: The optimal admission control policy is a state-dependent threshold-type, with threshold curve $A(x_{1},x_{3})$, such that a type-2 packet is admitted to queue 1 if and only if $x_{2}\leq A\left(x_{1},x_{3}\right)$ where $A\left(x_{1},x_{3}\right)=max\left\{x_{2}|w_{n}\left(x+e_{2}\right)-w_{n}\left(x\right)\geq c\right\}$.*
- ***Routing policy*** *: The optimal routing control policy is a state-dependent threshold-type, with threshold curves $R_{1}\left(x_{3}\right)$ and $R_{2}\left(x_{1},x_{3}\right)$, such that:*
  1. *Type-1 packet is routed to queue 2 if and only if $x_{2}=0$ and $x_{1}\geq R_{1}\left(x_{3}\right)$, where $R_{1}\left(x_{3}\right)=max\left\{x_{1}|w_{n}\left(x-e_{1}+e_{3}\right)-w_{n}\left(x\right)\geq c_{2}\right\}$.*
  2. *Type-2 packet is routed to queue 2 if and only if $x_{2}\geq R_{2}\left(x_{1},x_{3}\right)$, where $R_{2}\left(x_{1},x_{3}\right)=max\left\{x_{2}|w_{n}\left(x-e_{2}+e_{3}\right)-w_{n}\left(x\right)\geq 0\right\}$.*

The results of Theorem 1 also apply to the average reward criterion (see [57]). Hence, we will use the average reward criterion for all our numerical experiments as it has the advantage of not depending on the initial state. To prove the theorem, we will prove Properties 1–3. However, for ease of flow, we defer all mathematical proofs to the Appendix A.

## 5. Sensitivity Analysis of the Optimal Policy

**Figure 2:**
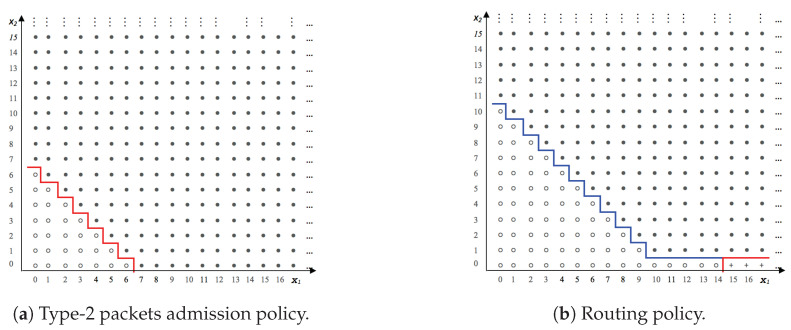
Optimal control policy.

In this section, we study the optimal control policy depicted in Theorem 1 and its sensitivity to the system parameters. We conduct extensive numerical experiments varying system parameters. As an illustration we consider a base case, where $\mu_{1}=0.35,\mu_{2}=0.45,$$\lambda_{1}\;=\;0.25,$$\lambda_{2}\;=\;0.25,$$r_{1}\;=\;80,$$r_{2}\;=\;60,r_{3}=30,h=0.7,c=5,c_{2}=0$. The optimal policy is computed using the value iteration algorithm of dynamic programming [58]. Convergence is obtained when the expected reward of successive iterations is within an accuracy of $10^{-5}$. The optimal admission control policy for this system is presented in Figure 2a. The optimal action is to reject type-2 packets in all states above the switching curve (above the red line) and to accept them in all states below the curve. Similarly, the optimal routing policy for the system is presented in Figure 2b. The optimal action is to route type-2 packets to queue 2 only in states above the switching curve (above the blue and red lines). In states below the switch-curve (below the blue line), no routing is allowed. Below the red line are the only states where type-1 packets are routed to queue 2 that is, when $x_{2}=0.$ We experimented with several system parameters and we obtained the same results in terms of the shape of the switching curves of the admission control policy and routing control policy.

**Figure 3:**
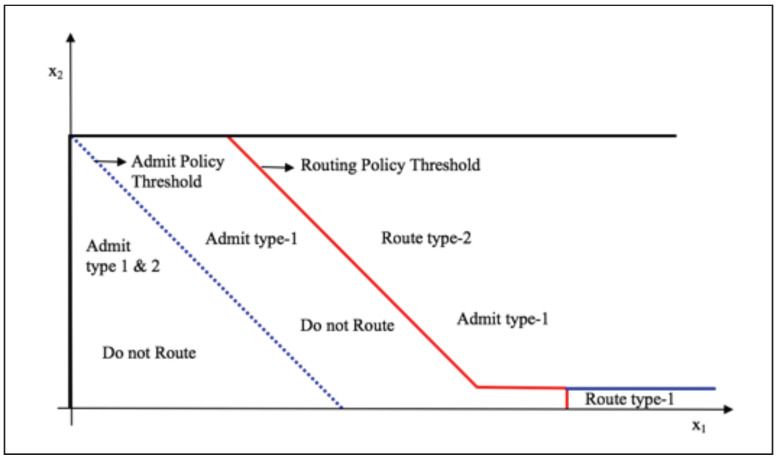
Admission and routing policy transient states for a given size of queue 2.

In Figure 3, we superpose both control policy curves where we note that the system gives priority to type-1 packets to be served at queue 1 by routing excess type-2 packets to queue 2. All numerical results gave a straight-line switching curve with slope of −1 in the $\left(x_{1},x_{2}\right)$ plane for a given $x_{3}$ packets in queue 2. However, proving this result analytically is untractable as it amounts to solving the optimality Equation (3) in closed form.

We further study the effect of the system parameters on the optimal average reward for various network load values, ρ∈{50%,75%,95%}. We isolate the effect of a particular system parameter by varying its value while holding the values of other system parameters constant.

**Figure 4:**
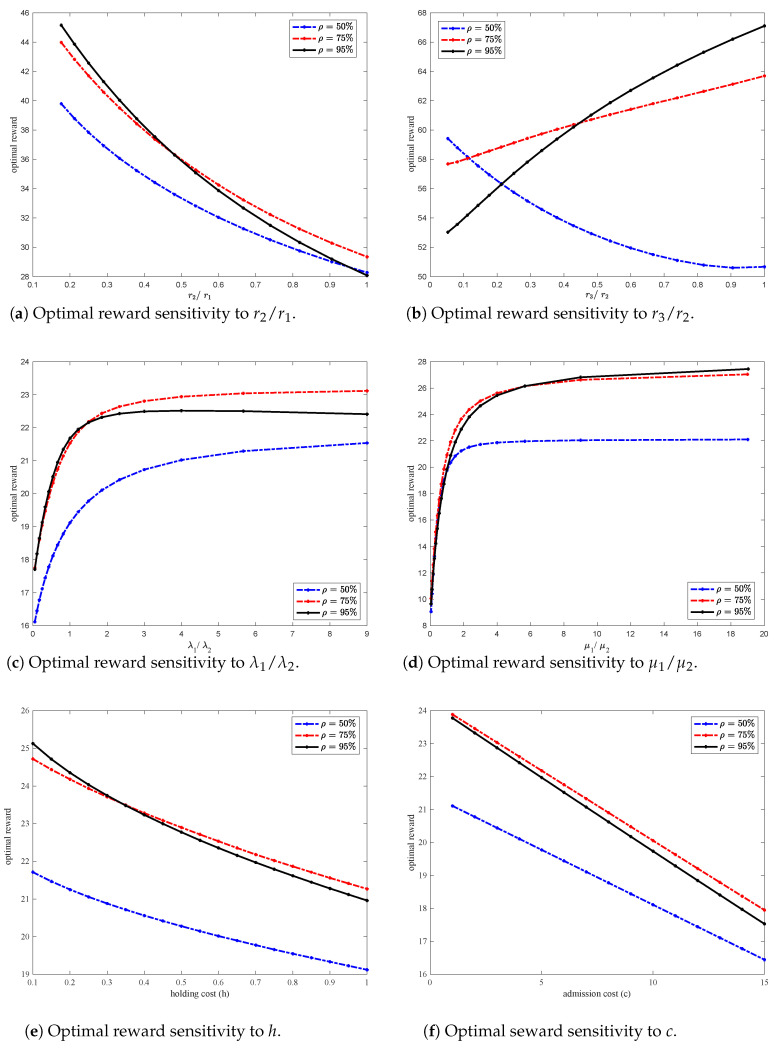
Optimal reward sensitivity to rewards $r_{1},r_{2},r_{3}$, service rates $\mu_{1},\mu_{2}$, arrival rates $\lambda_{1},\lambda_{2}$, holding cost *h* and admission cost *c*.

We study the effect of increasing reward $r_{2}$ while maintaining the sum of the rewards of $r_{1}$ and $r_{2}$ constant. Figure 4a shows that the optimal average reward decreases nonlinearly as the ratio $r_{2}/r_{1}$ increases. This behavior can be explained as follows. As $r_{2}$ increases, the incentive for packets to be routed to queue 2 decreases. Hence, queue 1 becomes overloaded and the overall holding cost eventually becomes high affecting the optimal average reward. Note however, that under heavy network load (ρ=95%), at a certain point, routing to queue 2 becomes inevitable causing lower reward compared to a system with lower network load (ρ=75%). This also explains the crossover of the curves corresponding to the optimal average reward curve for ρ=75% and the one for ρ=95% in the figure.

We further study the effect of increasing reward $r_{3}$ on the optimal average reward while maintaining the sum of the rewards of $r_{2}$ and $r_{3}$ constant. Figure 4b shows that the optimal average reward increases non-linearly as the ratio $r_{3}/r_{2}$ increases when the network load is high (ρ=75% and ρ=95%). This increase is due to the fact that as $r_{3}$ increases, packets in queue 1 have more incentive to be routed to queue 2 especially if queue 1 has type-1 packets. As type-2 packets are routed to queue 2, more space opens up in queue 1 for type-1 packets, and more type-2 packets are admitted, hence, the optimal average reward increases. Moreover, as the network load increases, routing admitted packets to queue 2 becomes sometimes necessary. Indeed, for higher network load (ρ=95%), the optimal average reward is increasing at a faster rate compared to the optimal average reward for a network with lower load of ρ=75%. For lower network load (ρ=50%) however, as $r_{3}$ increases the optimal average reward decreases nonlinearly. This can be explained as follows: as $r_{3}$ increases and if type-1 packets are in queue 1, then type-2 packets have more incentive to be routed to queue 2, and collect a lower reward hence, the optimal average reward decreases. The graphs generated in Figure 4a,b show results when $\mu_{1}=0.45,\mu_{2}=0.35,h=0.7,c=5,c_{2}=0$.

Figure 4c shows the sensitivity of the optimal average reward to the arrival rates while maintaining the sum of $\lambda_{1}$ and $\lambda_{2}$ constant. The figure shows that the optimal average reward initially increases at a high rate as $\lambda_{1}$ increases then the rate of increase slows down. The increase continues until queue 1 becomes congested to cause type-1 packets to be routed to queue 2, hence, collect a lower reward $r_{3}$, and decrease the optimal average reward. For high network load (ρ=95%) however, the optimal average reward eventually starts decreasing as the high load makes it necessary to increase routing packets to queue 2. Eventually, both queues saturate, leading to an increase in the holding cost, and a decrease in the optimal average reward. The graph generated in Figure 4c shows results for the following system parameters: $r_{1}=80,r_{2}=60,r_{3}=30,\mu_{1}=0.45,\mu_{2}=0.3,h=0.7,c=5$ and $c_{2}=0$.

In Figure 4d, we study the effect of the service rates $\mu_{1}$ and $\mu_{2}$ on the optimal average reward while maintaining the sum of $\mu_{1}$ and $\mu_{2}$ constant. As the ratio $\mu_{1}/\mu_{2}$ increases, the optimal average reward increases and eventually levels-off for all network loads considered (ρ∈{50%,75%,95%}). This can be explained as follows. As queue 1 service rate $\mu_{1}$ increases, queue 1’s length becomes shorter discouraging packets from being routed to queue 2. Hence, the optimal average reward increases. As $\mu_{1}$ continues to increase relative to $\mu_{2}$, less and less routing occurs eliminating the need for queue 2 which explains the leveling-off of the optimal average reward (since the arrival rates are held constant). In practice, however, achieving a high service rate to eliminate queue 2 is rather costly. The graph generated in Figure 4d shows results for the following system parameters: $r_{1}=80,r_{2}=60,r_{3}=30,\lambda_{1}=0.35,\lambda_{2}=0.3,h=0.7,c=5$ and $c_{2}=0$.

Figure 4e shows that the optimal average reward is nonlinearly decreasing in the holding cost *h*. Figure 4f shows that the optimal average reward is linearly decreasing in the admission cost *c*. These results are interesting and are worth exploiting in a future work in an attempt to get an analytical expression of the reward. The graphs generated in Figure 4e,f show results for the following system parameters: $r_{1}=80,r_{2}=60,r_{3}=30,\lambda_{1}=0.35,\lambda_{2}=0.3,c_{2}=0$ and various values of *h* and *c* respectively.

**Figure 5:**
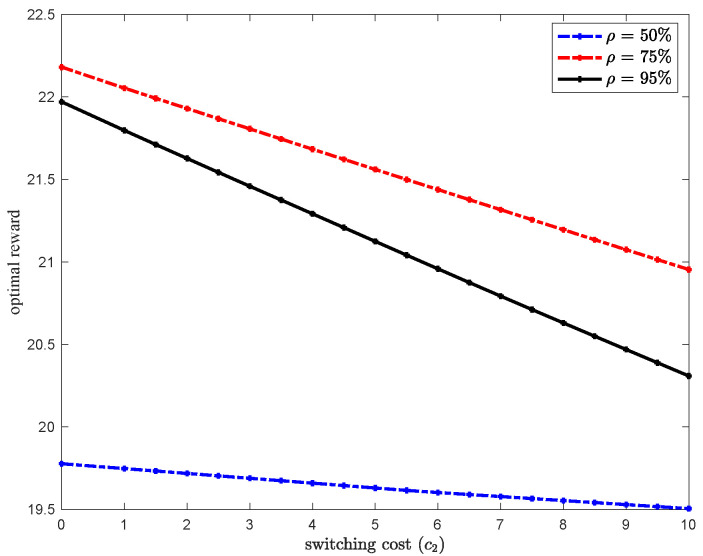
Optimal reward sensitivity to $c_{2}$.

Finally, we would like to note that even though our analysis focused on the case where the switching cost is zero ($c_{2}=0$), similar results are obtained for $c_{2}>0$. As an illustration, Figure 5, shows that the optimal reward linearly decreases in $c_{2}$. This result is expected as when the switching cost increases, type-1 packets have no incentive to be routed to queue 2. Moreover, as the network load increases, the optimal reward increases up to a point where both queues become congested. This explains why the optimal reward when ρ=75% is higher than the optimal reward when ρ=95% as $c_{2}$ increases. This also lead to the conclusion that there is an optimal load where the reward in maximized. The results in Figure 5 are obtained for system parameters: $r_{1}=80,r_{2}=60,r_{3}=30,$$\lambda_{1}\;=\;0.35,\lambda_{2}=0.3,h=0.7$ and c=5.

## 6. Heuristic Control Policy

It is well established that dynamic programming suffers from the curse of dimensionality. For our model in particular, the optimal policy is computationally untractable for systems with more than 2 types of packets (i.e., a state space with dimension greater than 3). Hence, it is too resource intensive to run on resource limited sensor devices. This motivated us to propose an efficient heuristic control policy that imitates the behavior of the optimal policy and is computationally much faster to obtain for the general case of *n* types of packets (see Algorithm 1). As such, we define the state of the system by the (n+1) dimensional vector $x=(x_{1},x_{2},\cdots ,x_{n},x_{n+1})$ where type-*i* packets take priority over type-*j* packets for i<j. The number of packets in queue 1 is represented by $x_{1}+x_{2}+\cdots +x_{n}$ where $x_{i}$ is the number of type-*i* packets while $x_{n+1}$ depicts the number of packets in queue 2. The heuristic control policy is characterized by 2(n−1) parameters: parameters $A_{i},i=2,\ldots ,n$ control the admission to queue 1, while parameters $R_{i},i=2,\ldots ,n$ control the routing to queue 2. Note that type-1 packets are always admitted to queue 1 (*i.e*, $A_{1}=\infty$). Furthermore, we have $A_{2}\geq A_{3}\geq \ldots \geq A_{n}$ and $R_{1}\geq R_{2}\geq \ldots \geq R_{n}\geq A_{2}$. We extend the costs and reward parameters as follows: *c* is the admission cost; *h* is the holding cost; $c_{i}$ is the switching cost for type-*i* packets ($c_{1}\geq c_{2}\geq \ldots \geq c_{n}$) and $r_{i},i\in \left\{1,2,\ldots ,n\right\}$ is the reward of type-*i* packet served at queue 1 ($r_{1}\geq r_{2}\geq \ldots \geq r_{n}$) and $r_{n+1}\left(<r_{n}\right)$ is the reward of packets served at queue 2.

At arrival of type-*i* packet, we use the following control policy where we define I(x)= the largest packet type i∈{1,…,n} such that $x_{i}>0$:   
**Algorithm 1.** Proposed heuristic control policy.**if**$\sum_{k=1}^{n}x_{k}<A_{i}$**then**      admit type-*i* packet to queue 1  **end if****if**$\sum_{k=1}^{n}x_{k}\geq A_{i}$ and I(x)≥i and $\sum_{k=1}^{n}x_{k}\geq R_{I(x)}$ **then**      admit type-*i* packet to queue 1 and route type-I(x) packet to queue 2  **else**     do no admit type-*i* packet  **end if**

In order to test the performance of the above proposed heuristic, we compare the reward generated by the heuristic to that of the optimal policy. We use the average reward criterion for this purpose. The average reward under the optimal policy is obtained using the following optimality equation:

(4) $$\begin{array}{c} w^{*}\left(x\right)+g^{*}=max\left\{T_{s}w\left(x\right),T_{r}w\left(x\right)\right\} \end{array}$$

where $g^{*}$ is the optimal average reward per transition (see [58]) and $w^{*}\left(x\right)$ is the optimal differential reward, $w\left(x\right),T_{s}$ and $T_{r}$ as defined in Section 3.2.

The average reward under the heuristic control policy (*H*) is defined using the following dynamic programming equation:

(5) $$\begin{array}{c} w^{H}\left(x\right)+g^{H}=-h\sum_{k=1}^{n+1}x_{k}+\sum_{k=1}^{n}\lambda_{i}T_{a_{i}}^{H}w\left(x\right)+\mu_{1}T_{1}^{H}w\left(x\right)+\mu_{2}T_{2}^{H}w\left(x\right). \end{array}$$

where $g^{H}$ is the average reward per transition under the heuristic control policy, $w^{H}\left(x\right)$ is the differential reward under the heuristic policy and $T_{a_{i}}^{H},T_{1}^{H}$ and $T_{2}^{H}$ are defined as follows:

$$\begin{array}{cc} & T_{a_{i}}^{H}w\left(x\right)=\left\{\begin{array}{cc} w(x+e_{i})-c\; & \mathrm{if}\sum_{k=1}^{n}x_{k}<A_{i}\\ w\left(x+e_{i}-e_{I(x)}+e_{n+1}\right)-c_{I(x)}\; & \mathrm{if}\sum_{k=1}^{n}x_{k}\geq A_{i},I\left(x\right)\geq i\;\mathrm{and}\;\sum_{k=1}^{n}x_{k}\geq R_{I(x)}\\ w(x)\; & \mathrm{otherwise} \end{array}\right. \end{array}$$

$$\begin{array}{cc} & T_{1}^{H}w\left(x\right)=\left\{\begin{array}{cc} w\left(x-e_{i}\right)+r_{i}\; & \mathrm{if}\;\exists \;i\;\mathrm{such}\;\mathrm{that}\;x_{i}>0\;\mathrm{and}\;x_{1}=x_{2}=\ldots =x_{i-1}=0\\ w(x)\; & \mathrm{otherwise} \end{array}\right. \end{array}$$

$$\begin{array}{cc} & T_{2}^{H}w\left(x\right)=\left\{\begin{array}{cc} w\left(x-e_{n+1}\right)+r_{i}\; & \mathrm{if}\;x_{n+1}>0\\ w(x)\; & \mathrm{otherwise} \end{array}\right. \end{array}$$

In the following, we compare the performance of the proposed heuristic control policy to the optimal control policy for the case of two types of packets. We examine the impact of a certain system variable by varying its value while maintaining all other variables constant. Similar to [59], we use as performance metric the reward Relative Deviation (RD) of the heuristic from the optimal. The RD, expressed in percentage, is defined as $RD=100\times \left(\psi^{*}-\psi^{H}\right)/\psi^{*}$ where $\psi^{*}$ denotes the average reward rate of the optimal control policy obtained by solving Equation (4), and $\psi^{H}$ denotes the average reward rate associated with the heuristic control policy obtained by solving Equation (5). Here, similar to the optimal policy, the expected reward is obtained using the value iteration algorithm with the same accuracy of $10^{-5}$.

Table 1 shows a sample of 100 randomly generated system parameter values used to compute the performance of the heuristic control policy. Based on these results, it is clear that the heuristic performs very well compared to the optimal policy. For a 95% confidence interval, the average RD is 1.40%±0.02% with a range of [0,4.29].

For a system with more than two types of packets, computing the optimal policy becomes untractable due to the exponential explosion of the number of states. However, computing the thresholds of this heuristic is orders of magnitude faster than computing the optimal policy. In fact, much of the computation required (i.e., computation of the system parameters) for the heuristic can be done off-line, and the real-time computation requires no more than a table lookup. The computation of the system parameters of the heuristic is approximately, and at worst equal to the number of parameters times the size of the square of the cardinality of the state space. Numerical results show that the heuristic performs very well compared to the optimal policy. For a 95% confidence interval, the average computation time is 0.045%±0.005% with a range of $\left[10^{-4},9\times 10^{-4}\right]$ over a sample of 100 cases.

Given that the computation time of the optimal policy scales exponentially in the state space, computing the optimal policy beyond two priority classes is untractable. For instance, consider a system with three types of packets. Even if we succeed to compute the optimal policy and the associated parameters, it will require a huge state dependent look-up table (five hyper-surfaces representing the state dependent thresholds). For the heuristic however, we will need to store only five static control parameters (i.e., two admission and three routing threshold parameters).

Finally, in practice, traffic flow (i.e., arrival rate λ) changes over time. Our model however, assumes a constant traffic flow ($\lambda_{i}$ for flow type-*i*). This is by no means a limitation of our model. In fact, this issue can be approached in one of two ways: either using a transient analysis which is well documented as being untractable especially in the context of an MDP framework; or computing different policy parameters off-line for each traffic flow. These parameters would be used for the particular traffic flow in effect during deployment.

## 7. Conclusions

In this paper, we considered an admission and routing control problem to address the issue of resource limitation in resource constrained networks (such as WSNs). We formulated the admission and routing control problem of two types of flows where one has a higher priority than the other, as a Markov decision problem. We characterized the optimal policy and showed that it is a state-dependent threshold type policy. Furthermore, we conducted extensive numerical experiments to gain more insight into the behavior of the optimal policy under different system parameters. Due to the computational challenges of the optimal policy (curse of dimensionality) which makes it untractable and too resource intensive to run on wireless devices, we proposed a heuristic that mimics the optimal control policy. Through extensive numerical results, we showed that the heuristic performs very well. It is also orders of magnitude faster than the optimal policy. Much of the required computation can be done off-line, and the real-time computation requires no more than a table lookup. We further generalized the heuristic for the case of a system with *n* types of flows (n≥2).

The results presented in this work provide a first step towards a better understanding of the structure of the optimal policy. There are several avenues for future research. In particular, it would be of interest to generalize the system to multi-server queues with more than two paths leading to the same destination. We expect the problem to become considerably more difficult with each additional feature and it is not clear if the optimal policy would be tractable. A clear extension of this work is to implement and test the proposed admission and routing control policy in real resource constrained network devices.

## Figures and Tables

**Table 1:** Heuristic performance compared to the optimal policy. RD is used as a measure of the Heuristic performance compared to the optimal policy.

$\mu_{1}$	$\mu_{2}$	$\lambda_{1}$	$\lambda_{2}$	$r_{1}$	$r_{2}$	$r_{3}$	RPD(%)
0.45	0.22	0.35	0.20	91	80	54	2.70
0.44	0.43	0.29	0.16	72	64	39	0.55
0.34	0.11	0.22	0.12	63	48	26	3.02
0.41	0.35	0.28	0.16	91	89	83	0.65
0.44	0.08	0.23	0.13	45	41	40	3.38
0.39	0.17	0.26	0.15	66	63	25	2.10
0.47	0.26	0.35	0.20	60	39	28	3.86
0.81	0.75	0.35	0.20	104	68	41	0.01
0.89	0.83	0.17	0.09	87	55	48	0.00
0.66	0.32	0.08	0.05	97	70	12	0.00
0.74	0.58	0.35	0.20	100	84	31	0.21
0.52	0.44	0.35	0.20	82	47	23	1.37
0.94	0.83	0.23	0.13	66	32	32	0.58
1.05	0.72	0.53	0.89	95	51	28	1.80
1.19	0.64	0.55	0.92	90	41	37	2.00
0.72	0.58	0.39	0.65	81	40	20	3.31
1.17	0.84	0.60	1.01	90	63	6	0.10
0.89	0.74	0.81	0.49	95	70	23	1.62
0.95	0.76	0.86	0.51	36	24	19	4.27
0.99	0.69	0.84	0.50	31	12	7	2.06
1.20	0.75	0.97	0.58	63	39	12	2.06
1.15	0.65	0.90	0.54	89	62	28	1.33
0.81	0.61	0.71	0.43	79	50	25	2.55
1.08	0.67	0.87	0.52	94	56	22	1.84
1.23	0.73	0.98	0.59	57	48	31	1.40
0.81	0.56	0.68	0.41	88	66	47	1.78
1.23	0.56	0.89	0.54	95	89	86	0.88
1.05	0.41	0.73	0.44	98	65	13	0.61
1.15	0.79	0.97	0.58	76	51	7	0.84
0.86	0.26	0.35	0.20	104	83	65	0.01
0.53	0.40	0.21	0.12	76	39	16	0.02
0.95	0.73	0.17	0.10	78	54	31	0.00
0.33	0.26	0.09	0.05	93	74	59	0.01
0.79	0.57	0.12	0.07	98	55	29	0.00
0.78	0.69	0.05	0.03	61	25	17	0.00
1.03	0.82	0.56	0.74	98	91	77	0.70
1.08	0.81	0.56	0.75	59	53	10	0.55
0.71	0.61	0.40	0.53	80	47	19	1.80
0.84	0.56	0.42	0.56	69	66	50	0.85
0.83	0.43	0.38	0.51	86	51	21	0.56
0.91	0.70	0.48	0.65	99	72	11	0.42
0.81	0.52	0.40	0.53	73	19	11	2.49
0.70	0.51	0.36	0.48	43	25	10	2.22
0.60	0.55	0.34	0.46	89	41	36	4.29
0.59	0.52	0.33	0.44	84	70	64	2.57
0.65	0.31	0.29	0.38	77	37	36	2.81
0.77	0.45	0.37	0.49	52	33	32	3.32
0.80	0.59	0.42	0.56	59	45	39	2.26
0.90	0.47	0.41	0.55	79	31	25	1.65
0.96	0.59	0.47	0.62	100	83	38	0.36
0.99	0.56	0.47	0.62	85	28	21	1.63
0.43	0.41	0.25	0.34	85	77	20	1.67
0.92	0.51	0.43	0.57	73	43	36	1.35
0.90	0.55	0.43	0.58	76	66	35	0.59
0.67	0.66	0.40	0.53	59	54	21	2.23
0.88	0.48	0.41	0.54	94	46	33	0.94
0.86	0.73	0.48	0.64	98	39	37	3.33
0.70	0.45	0.34	0.46	75	55	46	1.62
0.64	0.48	0.34	0.45	87	50	7	0.34
0.52	0.50	0.31	0.41	85	78	77	3.26
0.87	0.82	0.51	0.68	98	81	64	1.42
0.93	0.76	0.50	0.67	81	55	21	1.04
0.81	0.65	0.44	0.58	72	40	34	2.83
0.86	0.36	0.37	0.49	78	78	21	0.25
0.74	0.42	0.35	0.47	65	59	10	0.37
0.86	0.76	0.49	0.65	83	46	34	2.37
0.88	0.45	0.40	0.53	77	75	66	0.61
0.43	0.42	0.26	0.34	95	67	46	3.45
0.84	0.49	0.40	0.53	88	38	23	1.18
0.69	0.58	0.38	0.51	70	45	32	2.65
0.90	0.41	0.39	0.52	79	37	20	0.74
0.89	0.44	0.40	0.53	78	38	20	0.81
0.90	0.87	0.53	0.71	53	31	18	3.34
0.95	0.46	0.42	0.56	96	78	48	0.31
0.81	0.33	0.34	0.46	88	80	19	0.24
0.93	0.83	0.53	0.70	88	30	22	2.90
0.83	0.79	0.48	0.65	83	50	20	1.86
0.94	0.69	0.49	0.65	65	62	54	1.08
0.86	0.45	0.40	0.53	76	62	51	0.74
0.56	0.40	0.29	0.38	109	12	12	3.69
0.75	0.24	0.30	0.39	119	90	10	0.57
1.13	0.25	0.41	0.55	109	51	45	0.40
0.90	0.50	0.42	0.56	113	41	21	0.69
0.54	0.43	0.29	0.38	81	42	9	1.16
0.65	0.45	0.33	0.44	88	88	37	0.76
1.11	0.58	0.51	0.68	98	61	22	0.29
0.57	0.40	0.29	0.39	107	102	17	0.54
1.15	0.65	0.54	0.72	36	25	13	1.33
1.16	0.62	0.53	0.71	91	45	37	0.82
0.69	0.55	0.37	0.50	109	56	39	1.84
0.73	0.53	0.38	0.50	51	29	14	2.19
0.83	0.77	0.48	0.64	109	101	72	1.08
0.91	0.72	0.49	0.65	55	51	36	1.42
1.01	0.39	0.42	0.56	109	80	65	0.29
0.74	0.54	0.39	0.52	89	76	15	0.61
1.12	0.61	0.52	0.69	120	38	25	0.66
1.08	0.80	0.56	0.75	81	47	19	0.87
0.76	0.50	0.38	0.50	64	46	16	0.99
1.04	0.36	0.42	0.56	102	100	95	0.38
0.86	0.80	0.50	0.66	109	93	42	1.03

## Appendix A

In order to prove Theorem 1 we first state the following Lemma.

**Lemma** **A1.**
*Let V be the set of functions, v, defined on $R^{3}$ such that v satisfies Properties 1–3. Then $w_{n}\in V,\forall n\in Z^{+}$, where $Z^{+}$ is the set of non-negative integers.*

**Proof.** First, note that by adding Properties 2 and 3 we obtain Property 1. Therefore, it suffices to prove Properties 2 and 3. To prove these properties, we use induction on the remaining number of periods. The following is a sketch of the proof.

- *Step 1*: we observe that Properties 2 and 3 hold for n=0.
- *Step 2*: we assume 2 and 3 hold for some n≥0.
- *Step 3*: we prove that 2 and 3 hold for n+1.

We introduce the following notation:

$$\begin{array}{ccc} & \Delta_{i}w_{n}\left(x+e_{i}\right) & =w_{n}\left(x+e_{i}\right)-w_{n}\left(x\right)\\ & \Delta_{ij}w_{n}\left(x+e_{i}+e_{j}\right) & =w_{n}\left(x+e_{i}\right)-w_{n}\left(x\right) \end{array}$$

Hence, we can write

$$\begin{array}{ccc} & \max\{w_{n}\left(x+e_{i}\right),w_{n}\left(x\right)\} & =w_{n}\left(x\right)+\max\left\{w_{n}\left(x+e_{i}\right)-w_{n}\left(x\right),0\right\}\\ & & =w_{n}\left(x\right)+max\left\{\Delta_{i}w_{n}\left(x\right),0\right\} \end{array}$$

We show that $T_{s}$ and $T_{r}$ satisfy Properties 2 and 3 and thus satisfy Property 1.

$$\mathrm{Operator}\;T_{s}$$

Note that operators $T_{a_{1}},T_{1}$ and $T_{2}$ satisfy Properties 2 and 3 by induction as they do not involve any decision. Hence, we only need to show that $T_{a_{2}}$ satisfies Properties 2 and 3. Using the difference operator, we have:

$$\begin{array}{c} T_{a_{2}}w_{n-1}\left(x\right)=w_{n-1}\left(x\right)+\max\left\{\Delta_{2}w_{n-1}\left(x\right)-c,0\right\} \end{array}$$

To show that $T_{a_{2}}$ satisfies Property 2, let

$$\begin{array}{cc} \mathrm{Let}\;\;Q(x)= & T_{a_{2}}w_{n-1}\left(x+2e_{i}\right)-T_{a_{2}}w_{n-1}\left(x+e_{i}\right)-T_{a_{2}}w_{n-1}\left(x+e_{i}+e_{j}\right)+T_{a_{2}}w_{n-1}\left(x+e_{j}\right)\\ = & \left(\Delta_{i}w_{n-1}\left(x+e_{i}\right)-\Delta_{i}w_{n-1}\left(x+e_{j}\right)\right)+\max\left\{\Delta_{j}w_{n-1}\left(x+2e_{i}\right)-c,0\right\}-\max\left\{\Delta_{j}w_{n-1}\left(x+e_{i}\right)-c,0\right\}\\ \; & -\max\left(\Delta_{j}w_{n-1}\left(x+e_{i}+e_{j}\right)-c,0\right)+\max\left\{\Delta_{j}w_{n-1}\left(x+e_{j}\right)-c,0\right\} \end{array}$$

Using submodularity property, we infer that: $\Delta_{j}w_{n-1}\left(x+e_{i}\right)\geq \Delta_{j}w_{n-1}\left(x+e_{j}\right)\geq \Delta_{j}w_{n-1}\left(x+e_{i}+e_{j}\right)$ and

$$\Delta_{j}w_{n-1}\left(x+e_{i}\right)\geq \Delta_{j}w_{n-1}\left(x+2e_{i}\right)\geq \Delta_{j}w_{n-1}(x+e_{i}+e_{j}).$$

We prove the property for each case.

1. case 1: assume $\Delta_{j}w_{n-1}\left(x+e_{i}+e_{j}\right)-c\geq 0$
  $$\begin{array}{c} Q\left(x\right)=\Delta_{i}w_{n-1}\left(x+e_{i}+e_{j}\right)-\Delta_{i}w_{n-1}\left(x+2e_{j}\right)\leq 0,\;\;\;\;\mathrm{by}\;\mathrm{the}\;\mathrm{inductive}\;\mathrm{hypothesis}. \end{array}$$
2. case 2: assume $\Delta_{j}w_{n-1}\left(x+e_{j}\right)-c\geq 0\geq \Delta_{j}w_{n-1}\left(x+e_{i}+e_{j}\right)-c$ and $\Delta_{j}w_{n-1}\left(x+2e_{i}\right)-c\geq 0\geq \Delta_{j}w_{n-1}\left(x+e_{i}+e_{j}\right)-c$
  $$\begin{array}{cc} Q(x)= & \underset{\leq 0\;\mathrm{by}\;\mathrm{the}\;\mathrm{inductive}\;\mathrm{hypothesis}}{\underset{︸}{\Delta_{i}w_{n-1}\left(x+e_{i}+e_{j}\right)-\Delta_{i}w_{n-1}\left(x+2e_{j}\right)}}+\underset{\leq 0\;\mathrm{by}\;\mathrm{the}\;\mathrm{assumption}}{\underset{︸}{\Delta_{j}w_{n-1}\left(x+e_{i}+e_{j}\right)-c}}\leq 0 \end{array}$$
3. case 3: assume $\Delta_{j}w_{n-1}\left(x+e_{i}\right)-c\geq 0\geq \Delta_{j}w_{n-1}\left(x+e_{j}\right)-c$ $\geq \Delta_{j}w_{n-1}\left(x+e_{i}+e_{j}\right)-c$ and $\Delta_{j}w_{n-1}\left(x+2e_{i}\right)-c\geq 0$.
  $$\begin{array}{cc} Q(x)= & \underset{\leq 0\;\mathrm{by}\;\mathrm{the}\;\mathrm{inductive}\;\mathrm{hypothesis}}{\underset{︸}{\Delta_{i}w_{n-1}\left(x+e_{i}+e_{j}\right)-\Delta_{i}w_{n-1}\left(x+2e_{j}\right)}}+\underset{\leq 0\;\mathrm{by}\;\mathrm{the}\;\mathrm{assumption}}{\underset{︸}{\Delta_{j}w_{n-1}\left(x+e_{i}+e_{j}\right)-c-\Delta_{j}w_{n-1}\left(x+e_{j}\right)-c}}\leq 0 \end{array}$$
4. case 4: assume $\Delta_{j}w_{n-1}\left(x+e_{j}\right)-c\geq 0$ and $\Delta_{j}w_{n-1}\left(x+e_{i}\right)-c\geq 0\geq \Delta_{j}w_{n-1}\left(x+2e_{i}\right)-c$ $\geq \Delta_{j}w_{n-1}\left(x+e_{i}+e_{j}\right)-c$
  $$\begin{array}{cc} Q(x)= & \underset{\leq 0\;\mathrm{by}\;\mathrm{the}\;\mathrm{inductive}\;\mathrm{hypothesis}}{\underset{︸}{\Delta_{i}w_{n-1}\left(x+e_{i}+e_{j}\right)-\Delta_{i}w_{n-1}\left(x+2e_{j}\right)}}+\underset{\leq 0\;\mathrm{by}\;\mathrm{the}\;\mathrm{assumption}}{\underset{︸}{\Delta_{j}w_{n-1}\left(x+e_{i}+e_{j}\right)-c-\Delta_{j}w_{n-1}\left(x+2e_{i}\right)+c}}\leq 0 \end{array}$$
5. case 5: assume $\Delta_{j}w_{n-1}\left(x+e_{i}\right)-c\geq 0\geq \Delta_{j}w_{n-1}\left(x+e_{j}\right)-c$ $\geq \Delta_{j}w_{n-1}\left(x+e_{i}+e_{j}\right)-c$ and $\Delta_{j}w_{n-1}\left(x+e_{i}\right)-c\geq 0\geq \Delta_{j}w_{n-1}\left(x+2e_{i}\right)-c$ $\geq \Delta_{j}w_{n-1}\left(x+e_{i}+e_{j}\right)-c$
  $$\begin{array}{cc} Q(x)= & \underset{\leq 0\;\mathrm{by}\;\mathrm{the}\;\mathrm{inductive}\;\mathrm{hypothesis}}{\underset{︸}{\Delta_{i}w_{n-1}\left(x+e_{i}\right)-\Delta_{i}w_{n-1}\left(x+e_{j}\right)}}-\underset{\geq 0\;\mathrm{by}\;\mathrm{the}\;\mathrm{assumption}}{\underset{︸}{\Delta_{j}w_{n-1}\left(x+e_{i}\right)-c}}\leq 0 \end{array}$$
6. case 6: assume $0\geq \Delta_{j}w_{n-1}\left(x+e_{i}\right)-c\geq \Delta_{j}w_{n-1}\left(x+e_{j}\right)-c\geq \Delta_{j}w_{n-1}\left(x+e_{i}+e_{j}\right)-c$ and $0\geq \Delta_{j}w_{n-1}\left(x+e_{i}\right)-c\geq \Delta_{j}w_{n-1}\left(x+2e_{i}\right)-c\geq \Delta_{j}w_{n-1}\left(x+e_{i}+e_{j}\right)-c$
  $$\begin{array}{cc} Q(x)= & \Delta_{i}w_{n-1}\left(x+e_{i}\right)-\Delta_{i}w_{n-1}\left(x+e_{j}\right)\leq 0\;\;\mathrm{by}\;\mathrm{the}\;\mathrm{inductive}\;\mathrm{hypothesis}. \end{array}$$

Since the set V is closed under addition and multiplication by a scalar, it follows that $T_{s}$ satisfies Property 2. To show that $T_{a_{2}}$ satisfies Property 3, let

$$\begin{array}{cc} Q(x)= & T_{a_{2}}w_{n-1}\left(x+e_{i}+e_{j}\right)-T_{a_{2}}w_{n-1}\left(x+e_{j}\right)-T_{a_{2}}w_{n-1}\left(x+e_{i}\right)+T_{a_{2}}w_{n-1}\left(x\right)\\ = & \left(\Delta_{i}w_{n-1}\left(x+e_{j}\right)-\Delta_{i}w_{n-1}\left(x\right)\right)+\max\left\{\Delta_{j}w_{n-1}\left(x+e_{i}+e_{j}\right)-c,0\right\}-\max\left\{\Delta_{j}w_{n-1}\left(x+e_{j}\right)-c,0\right\}\\ \; & -\max\left\{\Delta_{j}w_{n-1}\left(x+e_{i}\right)-c,0\right\}+\max\left\{\Delta_{j}w_{n-1}\left(x\right)-c,0\right\} \end{array}$$

Using submodularity, we infer that $\Delta_{j}w_{n-1}\left(x\right)-c\geq \Delta_{j}w_{n-1}\left(x+e_{i}\right)-c\geq \Delta_{j}w_{n-1}\left(x+e_{j}\right)-c\geq \Delta_{j}w_{n-1}\left(x+e_{i}+e_{j}\right)-c$. In the following we prove the property for each case.

1. case 1: assume $\Delta_{j}w_{n-1}\left(x\right)-c\geq \Delta_{j}w_{n-1}\left(x+e_{i}\right)-c\geq \Delta_{j}w_{n-1}\left(x+e_{j}\right)-c\geq \Delta_{j}w_{n-1}\left(x+e_{i}+e_{j}\right)-c\geq 0$
  $$\begin{array}{cc} Q(x)= & \left(\Delta_{i}w_{n-1}\left(x+e_{j}\right)-\Delta_{i}w_{n-1}\left(x\right)\right)+\Delta_{j}w_{n-1}\left(x+e_{i}+e_{j}\right)-c-\Delta_{j}w_{n-1}\left(x+e_{j}\right)+c\\ \; & -\Delta_{j}w_{n-1}\left(x+e_{i}\right)+c+\Delta_{j}w_{n-1}\left(x\right)-c\\ = & \Delta_{i}w_{n-1}\left(x+2e_{j}\right)-\Delta_{i}w_{n-1}\left(x+e_{j}\right)\\ \leq & 0\;\;\mathrm{by}\;\mathrm{the}\;\mathrm{inductive}\;\mathrm{hypothesis} \end{array}$$
2. case 2: assume $\Delta_{j}w_{n-1}\left(x\right)-c\geq \Delta_{j}w_{n-1}\left(x+e_{i}\right)-c\geq \Delta_{j}w_{n-1}\left(x+e_{j}\right)-c\geq 0\geq \Delta_{j}w_{n-1}\left(x+e_{i}+e_{j}\right)-c$
  $$\begin{array}{cc} Q(x)= & \Delta_{i}w_{n-1}\left(x+2e_{j}\right)-\Delta_{i}w_{n-1}\left(x+e_{j}\right)-\Delta_{j}w_{n-1}\left(x+e_{i}+e_{j}\right)+c\\ = & \Delta_{i}w_{n-1}\left(x+2e_{j}\right)-\Delta_{i}w_{n-1}\left(x+e_{j}\right)-\Delta_{j}w_{n-1}\left(x+e_{i}+e_{j}\right)+c\\ = & -(\Delta_{j}w_{n-1}\left(x+e_{j}\right)-c)\\ \leq & 0\;\;\mathrm{by}\;\mathrm{the}\;\mathrm{assumption} \end{array}$$
3. case 3: assume $\Delta_{j}w_{n-1}\left(x\right)-c\geq \Delta_{j}w_{n-1}\left(x+e_{i}\right)-c\geq 0\geq \Delta_{j}w_{n-1}\left(x+e_{j}\right)-c\geq \Delta_{j}w_{n-1}\left(x+e_{i}+e_{j}\right)-c$
  $$\begin{array}{cc} Q(x)= & \underset{\leq 0\;\;\mathrm{from}\;\mathrm{case}\;2}{\underset{︸}{-(\Delta_{j}w_{n-1}\left(x+e_{j}\right)-c)}}\;+\underset{\leq 0\;\;\mathrm{by}\;\mathrm{the}\;\mathrm{assumption}}{\underset{︸}{\Delta_{j}w_{n-1}\left(x+e_{j}\right)-c}}\;\;\;\;=0 \end{array}$$
4. case 4: assume $\Delta_{j}w_{n-1}\left(x\right)-c\geq 0\geq \Delta_{j}w_{n-1}\left(x+e_{i}\right)-c\geq \Delta_{j}w_{n-1}\left(x+e_{j}\right)-c\geq \Delta_{j}w_{n-1}\left(x+e_{i}+e_{j}\right)-c$
  $$\begin{array}{cc} Q(x)= & \underset{\mathrm{Per}\;\mathrm{case}\;3}{\underset{︸}{0}}+\underset{\leq 0\;\;\mathrm{by}\;\mathrm{the}\;\mathrm{assumption}}{\underset{︸}{\Delta_{j}w_{n-1}\left(x+e_{i}\right)-c}}\;\leq 0 \end{array}$$
5. case 5: assume $0\geq \Delta_{j}w_{n-1}\left(x\right)-c\geq \Delta_{j}w_{n-1}\left(x+e_{i}\right)-c\geq \Delta_{j}w_{n-1}\left(x+e_{j}\right)-c\geq \Delta_{j}w_{n-1}\left(x+e_{i}+e_{j}\right)-c$
  $$\begin{array}{cc} Q(x)= & (\Delta_{i}w_{n-1}\left(x+e_{j}\right)-\Delta_{i}w_{n-1}\left(x\right))\leq 0\;\mathrm{by}\;\mathrm{the}\;\mathrm{inductive}\;\mathrm{hypothesis} \end{array}$$

Since the set V is closed under addition and multiplication by a scalar, it follows that $T_{s}$ satisfies Property 3.

$$\mathrm{Operator}T_{r}$$

$$\begin{array}{cc} & T_{r}w_{n-1}\left(x\right)=\left\{\begin{array}{cc} max\{w_{n-1}\left(x-e_{1}+e_{3}\right)-c_{2},w_{n-1}\left(x\right)\}\; & \;\;\;\mathrm{if}\;x_{2}=0,\;x_{1}\geq 0,\;\mathrm{route}\;\mathrm{type}\;1\\ max\{w_{n-1}\left(x-e_{2}+e_{3}\right),w_{n-1}\left(x\right)\}\; & \;\;\;\mathrm{if}\;x_{2}>0,\;\mathrm{route}\;\mathrm{type}\;2 \end{array}\right. \end{array}$$

To show that $T_{r}$ satisfies Property 2, we need to prove that

$$\begin{array}{c} T_{r}w_{n-1}\left(x+2e_{i}\right)-T_{r}w_{n-1}\left(x+e_{i}\right)-T_{r}w_{n-1}\left(x+e_{i}+e_{j}\right)+T_{r}w_{n-1}\left(x+e_{j}\right)\leq 0 \end{array}$$

For simplicity we prove the property for i=1 and j=2. The rest of the properties follow the same procedure.

1. $x_{2}=0,\;x_{1}\geq 0$
  (A1) $$\begin{array}{cc} T_{r}w_{n-1}\left(x\right)= & w_{n-1}\left(x\right)+max\left\{\Delta_{-13}w_{n-1}\left(x\right)-c_{2},0\right\} \end{array}$$
  $$\begin{array}{cc} \mathrm{Let}\;\;Q(x)= & T_{r}w_{n-1}\left(x+2e_{1}\right)-T_{r}w_{n-1}\left(x+e_{1}\right)-T_{r}w_{n-1}\left(x+e_{1}+e_{2}\right)+T_{r}w_{n-1}\left(x+e_{2}\right)\\ = & w_{n-1}\left(x+2e_{1}\right)-w_{n-1}\left(x+e_{1}\right)-w_{n-1}\left(x+e_{1}+e_{2}\right)+w_{n-1}\left(x+e_{2}\right)\\ \; & +max\left\{\Delta_{-13}w_{n-1}\left(x+2e_{1}\right)-c_{2},0\right\}-max\left\{\Delta_{-13}w_{n-1}\left(x+e_{1}\right)-c_{2},0\right\}\\ \; & -max\left\{\Delta_{-13}w_{n-1}\left(x+e_{1}+e_{2}\right)-c_{2},0\right\}+max\left\{\Delta_{-13}w_{n-1}\left(x+e_{2}\right)-c_{2},0\right\} \end{array}$$
  Note that, by Property 2 the following inequalities hold.
  $\Delta_{-13}w_{n-1}\left(x+e_{1}\right)-c_{2}\geq \Delta_{-13}w_{n-1}\left(x+e_{1}+e_{2}\right)-c_{2}\geq \Delta_{-13}w_{n-1}\left(x+2e_{1}\right)-c_{2}\geq \Delta_{-13}w_{n-1}\left(x+e_{2}\right)-c_{2}$
  We prove the property for each case.
  (a) case 1: assume $\Delta_{-13}w_{n-1}\left(x+e_{1}\right)-c_{2}\geq \Delta_{-13}w_{n-1}\left(x+e_{1}+e_{2}\right)-c_{2}\geq \Delta_{-13}w_{n-1}\left(x+2e_{1}\right)-c_{2}\geq \Delta_{-13}w_{n-1}\left(x+e_{2}\right)-c_{2}\geq 0.$
    $$\begin{array}{cc} Q(x)= & w_{n-1}\left(x+2e_{1}\right)-w_{n-1}\left(x+e_{1}\right)-w_{n-1}\left(x+e_{1}+e_{2}\right)+w_{n-1}\left(x+e_{2}\right)\\ \; & +\Delta_{-13}w_{n-1}\left(x+2e_{1}\right)-\Delta_{-13}w_{n-1}\left(x+e_{1}\right)-\Delta_{-13}w_{n-1}\left(x+e_{1}+e_{2}\right)+\Delta_{-13}w_{n-1}\left(x+e_{2}\right)\\ = & \Delta_{1}w_{n-1}\left(x+e_{3}\right)-\Delta_{1}w_{n-1}\left(x-e_{1}+e_{2}+e_{3}\right)\\ \leq & 0\;\mathrm{by}\;\mathrm{the}\;\mathrm{inductive}\;\mathrm{hypothesis} \end{array}$$
  (b) case 2: assume $\Delta_{-13}w_{n-1}\left(x+e_{1}\right)-c_{2}\geq \Delta_{-13}w_{n-1}\left(x+e_{1}+e_{2}\right)-c_{2}\geq \Delta_{-13}w_{n-1}\left(x+2e_{1}\right)-c_{2}\geq 0\geq \Delta_{-13}w_{n-1}\left(x+e_{2}\right)-c_{2}.$
    $$\begin{array}{cc} Q(x)= & \underset{\mathrm{From}\;\mathrm{case}\;1}{\underset{︸}{\Delta_{1}w_{n-1}\left(x+e_{3}\right)-\Delta_{1}w_{n-1}\left(x-e_{1}+e_{2}+e_{3}\right)}}-\left(\Delta_{-13}w_{n-1}\left(x+e_{2}\right)-c_{2}\right)\\ \; & \mathrm{using}\;\mathrm{the}\;\mathrm{assumption},\;\Delta_{-13}w_{n-1}\left(x+e_{1}+e_{2}\right)-c_{2}\geq \Delta_{-13}w_{n-1}\left(x+2e_{1}\right)-c_{2},\\ \; & \mathrm{and}\;\mathrm{after}\;\mathrm{simplification},\;\mathrm{we}\;\mathrm{can}\;\mathrm{express}\;Q(x)\;\mathrm{as},\\ Q(x)\leq & \;\;\underset{\leq 0\;\mathrm{by}\;\mathrm{concavity}\;\mathrm{Property}\;2}{\underset{︸}{\;\;\Delta_{1}w_{n-1}\left(x+e_{1}\right)-\Delta_{1}w_{n-1}\left(x+e_{2}\right)\;\;}}\;-\underset{\geq 0\;\mathrm{by}\;\mathrm{assumption}}{\underset{︸}{\;\;(\Delta_{-13}w\left(x+e_{1}\right)-c_{2})\;\;\;}}\\ \leq & 0 \end{array}$$
  (c) case 3: assume $\Delta_{-13}w_{n-1}\left(x+e_{1}\right)-c_{2}\geq \Delta_{-13}w_{n-1}\left(x+e_{1}+e_{2}\right)-c_{2}\geq 0\geq \Delta_{-13}w_{n-1}\left(x+2e_{1}\right)-c_{2}\geq \Delta_{-13}w_{n-1}\left(x+e_{2}\right)-c_{2}.$
    $$\begin{array}{cc} Q(x)\leq & \;\;\underset{\leq 0\;\mathrm{from}\;\mathrm{previous}\;\mathrm{case}}{\underset{︸}{\;\;\Delta_{1}w_{n-1}\left(x+e_{1}\right)-\Delta_{1}w_{n-1}\left(x+e_{2}\right)-\left(\Delta_{-13}w_{n-1}\left(x+e_{1}\right)-c_{2}\right)}}-\left(\Delta_{-13}w_{n-1}\left(x+2e_{1}\right)-c_{2}\right)\\ \leq & \;\;\underset{\leq 0\;\mathrm{from}\;\mathrm{previous}\;\mathrm{case}}{\underset{︸}{\;\;\Delta_{1}w_{n-1}\left(x+e_{1}\right)-\Delta_{1}w_{n-1}\left(x+e_{2}\right)-\left(\Delta_{-13}w_{n-1}\left(x+e_{1}\right)-c_{2}\right)}}-\underset{\leq \Delta_{-13}w\left(x+e_{1}\right)-c_{2}\;\mathrm{by}\;\mathrm{assumption}}{\underset{︸}{\;\;(\Delta_{-13}w_{n-1}\left(x+2e_{1}\right)-c_{2})}}\\ \leq & \;\;\Delta_{i}w_{n-1}\left(x+e_{1}\right)-\Delta_{i}w_{n-1}\left(x+e_{2}\right)-2\left(\Delta_{-13}w_{n-1}\left(x+e_{1}\right)-c_{2}\right)\\ \leq & 0 \end{array}$$
  (d) case 4: assume $\Delta_{-13}w_{n-1}\left(x+e_{1}\right)-c_{2}\geq 0\geq \Delta_{-13}w_{n-1}\left(x+e_{1}+e_{2}\right)-c_{2}\geq \Delta_{-13}w_{n-1}\left(x+2e_{1}\right)-c_{2}\geq \Delta_{-13}w_{n-1}\left(x+e_{2}\right)-c_{2}.$
    $$\begin{array}{cc} Q(x)\leq & \underset{\leq 0\;\mathrm{from}\;\mathrm{previous}\;\mathrm{case}}{\underset{︸}{\;\;\Delta_{1}w_{n-1}\left(x+e_{1}\right)-\Delta_{1}w_{n-1}\left(x+e_{2}\right)-2\left(\Delta_{-13}w_{n-1}\left(x+e_{1}\right)-c_{2}\right)}}+\underset{\leq 0\;\mathrm{by}\;\mathrm{assumption}}{\underset{︸}{\Delta_{-13}w_{n-1}\left(x+e_{1}+e_{3}\right)+c_{2}}}\\ \leq & 0 \end{array}$$
  (e) case 5: assume $0\geq \Delta_{-13}w_{n-1}\left(x+e_{1}\right)-c_{2}\geq \Delta_{-13}w_{n-1}\left(x+e_{1}+e_{2}\right)-c_{2}\geq \Delta_{-13}w_{n-1}\left(x+2e_{1}\right)-c_{2}\geq \Delta_{-13}w_{n-1}\left(x+e_{2}\right)-c_{2}.$
    $$\begin{array}{cc} Q(x)\leq & \underset{\leq 0\;\mathrm{from}\;\mathrm{previous}\;\mathrm{case}}{\underset{︸}{\;\;\Delta_{1}w_{n-1}\left(x+e_{1}\right)-\Delta_{1}w_{n-1}\left(x+e_{2}\right)-2\left(\Delta_{-13}w_{n-1}\left(x+e_{1}\right)-c_{2}\right)+\Delta_{-13}w_{n-1}\left(x+e_{1}+e_{3}\right)+c_{2}}}\\ \; & +\underset{\leq 0\;\mathrm{by}\;\mathrm{assumption}}{\underset{︸}{\Delta_{-13}w_{n-1}\left(x+e_{1}\right)+c_{2}}}\\ \leq & 0 \end{array}$$
2. $x_{2}>0$
  (A2) $$\begin{array}{cc} T_{r}w_{n-1}\left(x\right)= & max\{w_{n-1}\left(x-e_{j}+e_{3}\right),w_{n-1}\left(x\right)\}\\ = & w_{n-1}\left(x\right)+max\left\{\Delta_{-23}w_{n-1}\left(x\right),0\right\} \end{array}$$
  Note that $T_{r}\left(x\right)$ given in Equation (A2) is a special case of $T_{r}\left(x\right)$ expression given in Equation (A1) where $c_{2}=0$ and $e_{i}$ is substituted by $e_{j}$, therefore the proof of Property 2 follows.

In order to prove that $T_{r}$ satisfies Property 3, we need to prove that

$$\begin{array}{c} T_{r}w_{n-1}\left(x+e_{i}+e_{j}\right)-T_{r}w_{n-1}\left(x+e_{j}\right)-T_{r}w_{n-1}\left(x+e_{i}\right)+T_{r}w_{n-1}\left(x\right)\leq 0. \end{array}$$

For simplicity we prove the property for i=1 and j=2. The rest of the properties follow the same procedure.

1. $x_{2}=0,\;x_{1}\geq 0$
  (A3) $$\begin{array}{c} T_{r}w_{n-1}\left(x\right)=w_{n-1}\left(x\right)+max\left\{\Delta_{-13}w_{n-1}\left(x\right)-c_{2},0\right\} \end{array}$$
  $$\begin{array}{cc} Q(x)= & T_{r}w_{n-1}\left(x+e_{1}+e_{2}\right)-T_{r}w_{n-1}\left(x+e_{2}\right)-T_{r}w_{n-1}\left(x+e_{1}\right)+T_{r}w_{n-1}\left(x\right)\leq 0\\ = & w_{n-1}\left(x+e_{1}+e_{2}\right)-w_{n-1}\left(x+e_{2}\right)-w_{n-1}\left(x+e_{1}\right)+w_{n-1}\left(x\right)\\ \; & +max\left\{\Delta_{-13}w_{n-1}\left(x+e_{1}+e_{2}\right)-c_{2},0\right)\}-max\left\{\Delta_{-13}w_{n-1}\left(x+e_{2}\right)-c_{2},0\right)\}\\ \; & -max\left\{\Delta_{-13}w_{n-1}\left(x+e_{1}\right)-c_{2},0\right\}+max\left\{\Delta_{-13}w_{n-1}\left(x\right)-c_{2},0\right\} \end{array}$$
  Note that, by Property 2 the following inequalities hold. $\Delta_{-13}w_{n-1}\left(x+e_{1}+e_{2}\right)-c_{2}\geq \Delta_{-13}w_{n-1}\left(x+e_{2}\right)-c_{2}\geq \Delta_{-13}w_{n-1}\left(x+e_{1}\right)-c_{2}\geq \Delta_{-13}w_{n-1}\left(x\right)-c_{2}.$. We prove the property for each case.
  (a) case 1: assume $\Delta_{-13}w_{n-1}\left(x+e_{1}+e_{2}\right)-c_{2}\geq \Delta_{-13}w_{n-1}\left(x+e_{2}\right)-c_{2}\geq \Delta_{-13}w_{n-1}\left(x+e_{1}\right)-c_{2}\geq \Delta_{-13}w_{n-1}\left(x\right)-c_{2}\geq 0$
    $$\begin{array}{cc} Q(x)= & T_{r}w_{n-1}\left(x+e_{1}+e_{2}\right)-T_{r}w_{n-1}\left(x+e_{2}\right)-T_{r}w_{n-1}\left(x+e_{1}\right)+T_{r}w\left(x\right)\\ = & w_{n-1}\left(x+e_{1}+e_{2}\right)-w_{n-1}\left(x+e_{2}\right)-w_{n-1}\left(x+e_{1}\right)+w_{n-1}\left(x\right)\\ \; & +\left(w_{n-1}\left(x+e_{2}+e_{3}\right)-w_{n-1}\left(x+e_{1}+e_{2}\right)-c_{2}\right)-\left(w_{n-1}\left(x-e_{1}+e_{2}+e_{3}\right)-w_{n-1}\left(x+e_{2}\right)-c_{2}\right)\\ \; & -\left(w_{n-1}\left(x+e_{3}\right)-w_{n-1}\left(x+e_{1}\right)-c_{2}\right)+\left(w_{n-1}\left(x-e_{1}+e_{3}\right)-w_{n-1}\left(x\right)-c_{2}\right)\\ = & \Delta_{1}w_{n-1}\left(x-e_{1}+e_{2}+e_{3}\right)-\Delta_{1}w_{n-1}\left(x-e_{1}+e_{3}\right)\\ \leq & 0\;\mathrm{by}\;\mathrm{the}\;\mathrm{inductive}\;\mathrm{proposition}\;\mathrm{of}\;\mathrm{Property}\; \end{array}$$
  (b) case 2: assume $\Delta_{-13}w_{n-1}\left(x+e_{1}+e_{2}\right)-c_{2}\geq \Delta_{-13}w_{n-1}\left(x+e_{2}\right)-c_{2}\geq \Delta_{-13}w_{n-1}\left(x+e_{1}\right)-c_{2}\geq 0\geq \Delta_{-13}w_{n-1}\left(x\right)-c_{2}.$
    $$\begin{array}{cc} Q(x)= & T_{r}w_{n-1}\left(x+e_{1}+e_{2}\right)-T_{r}w_{n-1}\left(x+e_{2}\right)-T_{r}w_{n-1}\left(x+e_{1}\right)+T_{r}w_{n-1}\left(x\right)\\ = & \Delta_{1}w_{n-1}\left(x-e_{1}+e_{2}+e_{3}\right)-\Delta_{1}w_{n-1}\left(x-e_{1}+e_{3}\right)-\left(\Delta_{-13}w_{n-1}\left(x\right)-c_{2}\right)\\ \; & \mathrm{Per}\;\mathrm{assumption}\;w_{n-1}\left(x+e_{2}+e_{3}\right)-w_{n-1}\left(x-e_{1}+e_{2}+e_{3}\right)\geq w_{n-1}\left(x+e_{1}+e_{2}\right)-w_{n-1}\left(x+e_{2}\right)\\ Q(x)\leq & w_{n-1}\left(x+e_{1}+e_{2}\right)-w_{n-1}\left(x+e_{2}\right)+w_{n-1}\left(x\right)-\underset{\geq w_{n-1}\left(x+e_{1}\right)\;\mathrm{by}\;\mathrm{the}\;\mathrm{assumption}}{\underset{︸}{\left(w_{n-1}\left(x+e_{3}\right)-c_{2}\right)}}\\ \leq & w_{n-1}\left(x+e_{1}+e_{2}\right)-w_{n-1}\left(x+e_{2}\right)+w_{n-1}\left(x\right)-w_{n-1}\left(x+e_{1}\right)\\ = & \Delta_{1}w_{n-1}\left(x+e_{2}\right)-\Delta_{1}w_{n-1}\left(x\right)\\ \leq & 0\;\mathrm{by}\;\mathrm{the}\;\mathrm{inductive}\;\mathrm{hypothesis} \end{array}$$
  (c) case 3: assume $\Delta_{-13}w_{n-1}\left(x+e_{1}+e_{2}\right)-c_{2}\geq \Delta_{-13}w_{n-1}\left(x+e_{2}\right)-c_{2}\geq 0\geq \Delta_{-13}w_{n-1}\left(x+e_{1}\right)-c_{2}\geq \Delta_{-13}w_{n-1}\left(x\right)-c_{2}.$
    $$\begin{array}{cc} Q(x)= & \underset{\leq 0\;\mathrm{from}\;\mathrm{previous}\;\mathrm{case}}{\underset{︸}{\Delta_{1}w_{n-1}\left(x+e_{2}\right)-\Delta_{1}w_{n-1}\left(x\right)}}+\underset{\leq 0\;\mathrm{by}\;\mathrm{assumption}}{\underset{︸}{\left(\Delta_{-13}w_{n-1}\left(x+e_{i}\right)-c_{2}\right)}}\\ \leq & 0 \end{array}$$
  (d) case 4: assume $\Delta_{-13}w_{n-1}\left(x+e_{1}+e_{2}\right)-c_{2}\geq 0\geq \Delta_{-13}w_{n-1}\left(x+e_{2}\right)-c_{2}\geq \Delta_{-13}w_{n-1}\left(x+e_{1}\right)-c_{2}\geq \Delta_{-13}w_{n-1}\left(x\right)-c_{2}.$
    $$\begin{array}{cc} Q_{4}\left(x\right)= & \underset{\leq 0\;\mathrm{from}\;\mathrm{previous}\;\mathrm{case}}{\underset{︸}{\Delta_{1}w_{n-1}\left(x+e_{2}\right)-\Delta_{1}w_{n-1}\left(x\right)+\left(\Delta_{-13}w_{n-1}\left(x+e_{i}\right)-c_{2}\right)}}+\underset{\leq 0\;\mathrm{by}\;\mathrm{assumption}}{\underset{︸}{\left(\Delta_{-13}w_{n-1}\left(x+e_{j}\right)-c_{2}\right)}}\\ \leq & 0 \end{array}$$
  (e) case 5: assume $0\geq \Delta_{-13}w_{n-1}\left(x+e_{1}+e_{2}\right)-c_{2}\geq \Delta_{-13}w_{n-1}\left(x+e_{2}\right)-c_{2}\geq \Delta_{-13}w_{n-1}\left(x+e_{1}\right)-c_{2}\geq \Delta_{-13}w_{n-1}\left(x\right)-c_{2}.$
    $$\begin{array}{cc} Q\left(x\right)=\Delta_{1}w_{n-1}\left(x+e_{2}\right)-\Delta_{1}w_{n-1}\left(x\right)\leq & 0\;\mathrm{by}\;\mathrm{the}\;\mathrm{inductive}\;\mathrm{hypothesis}. \end{array}$$
2. For $x_{2}>0$
  (A4) $$\begin{array}{cc} T_{r}w_{n-1}\left(x\right)= & max\{w_{n-1}\left(x-e_{2}+e_{3}\right),w_{n-1}\left(x\right)\}\\ = & w_{n-1}\left(x\right)+max\left\{\Delta_{-23}w_{n-1}\left(x\right),0\right\} \end{array}$$
  Note that the expression of $T_{r}\left(x\right)$ given in Equation (A4) is a special case of the one of $T_{r}\left(x\right)$ given in Equation (A3) where $c_{2}=0$ and $e_{i}$ is substituted by $e_{j}$, therefore $T_{r}\left(x\right)$ satisfies Property 3. Finally, it is straightforward (using the induction argument) to show that $w_{n}\left(x\right)=max\left(T_{s}w_{n-1}\left(x\right),T_{r}w_{n-1}\left(x\right)\right),\mathrm{for}\;\;x_{1}+x_{2}>0$ satisfies Properties 2 and 3, hence satisfies Property 1. This completes the proof of Lemma A1.

 □

**Proof** (Proof of Theorem 1). For a given *n* we obtain an optimal policy $\Pi_{n}$. It can be shown that there exists an optimal stationary policy $\Pi^{*}$ such that $\Pi^{*}=\lim_{n\to \infty }\Pi_{n}$. This is true because $w_{n}\left(x\right)$ converges to an optimal time-independent discounted reward, *w*, for any α>0 (see [58]). The admission policy structure is due to Property 1. Indeed the latter indicates that the difference $w_{n}\left(x+e_{2}\right)-w_{n}\left(x\right)$ is non increasing in $x_{2}$. Thus $w_{n}\left(x\right)$ has a unique maximum at $A(x_{1},x_{3})$ as defined in the theorem. The routing policy structure for type-2 packets is due to Property 2. The latter indicates that the difference $w_{n}\left(x-e_{2}+e_{3}\right)-w_{n}\left(x\right)$ is non decreasing in $x_{2}$. Therefore, there exists a unique threshold $R_{2}\left(x_{1},x_{3}\right)$. Similarly, for type-1 packets, Property 3 implies that there exists a unique threshold $R_{1}\left(x_{3}\right)$ (since $x_{2}=0$ in this case). $R_{1}\left(x_{3}\right)$ and $R_{2}\left(x_{1},x_{3}\right)$ are defined in the theorem. This completes the proof of the theorem. □

## References

[1] Puccinelli D., Haenggi M. (2005). Wireless sensor networks: Applications and challenges of ubiquitous sensing. IEEE Circuits Syst. Mag..

[2] Rabby M.K.M., Alam M.S. (2019). A priority based energy harvesting scheme for charging embedded sensor nodes in wireless body area networks. PLoS ONE.

[3] Anguita D., Brizzolara D., Parodi G. Building an Underwater Wireless Sensor Network Based on Optical: Communication: Research Challenges and Current Results. Proceedings of the 2009 Third International Conference on Sensor Technologies and Applications.

[4] Hassan J. Future of Applications In Mobile wireless Sensor Networks. Proceedings of the 2018 1st International Conference on Computer Applications Information Security (ICCAIS).

[5] Nishikawa Y., Sasamura T., Ishizuka Y., Sugimoto S., Iwasaki S., Wang H., Fujishima T., Fujimoto T., Yamashita K., Suzuki T. Design of stable wireless sensor network for slope monitoring. Proceedings of the 2018 IEEE Topical Conference on Wireless Sensors and Sensor Networks (WiSNet).

[6] Sun Z., Akyildiz I.F. Connectivity in Wireless Underground Sensor Networks. Proceedings of the 2010 7th Annual IEEE Communications Society Conference on Sensor, Mesh and Ad Hoc Communications and Networks (SECON).

[7] Bogdanoski M., Shuminoski T., Risteski A. (2013). Analysis of the SYN flood DoS attack. Int. J. Comput. Netw. Inf. Secur..

[8] Oncioiu R., Simion E. Approach to Prevent SYN Flood DoS Attacks in Cloud. Proceedings of the 2018 International Conference on Communications (COMM).

[9] Eberhardt J., Tai S. On or Off the Blockchain? Insights on Off-Chaining Computation and Data. Proceedings of the European Conference on Service-Oriented and Cloud Computing.

[10] Gupta M.K., Hemachandra N., Venkateswaran J. (2018). Some parametrized dynamic priority policies for 2-class M/G/1 queues: Completeness and applications. arXiv.

[11] Dimitris B., Niño-Mora J. (1999). Optimization of Multiclass Queueing Networks with Changeover Times via the Achievable Region Approach: Part II, the Multi-Station Case. Math. Oper. Res..

[12] Hassin R., Puerto J., Fernandez F.R. (2009). The use of relative priorities in optimizing the performance of a queueing system. Eur. J. Oper. Res..

[13] Gupta M.K., Hemachandra N. On 2-moment completeness of non pre-emptive, non anticipative work conserving scheduling policies in some single class queues. Proceedings of the 2015 13th International Symposium on Modeling and Optimization in Mobile, Ad Hoc, and Wireless Networks (WiOpt).

[14] Rawal A., Kavitha V., Gupta M.K. Optimal surplus capacity utilization in polling systems via fluid models. Proceedings of the 2014 12th International Symposium on Modeling and Optimization in Mobile, Ad Hoc, and Wireless Networks (WiOpt).

[15] Li C., Neely M.J. Delay and rate-optimal control in a multi-class priority queue with adjustable service rates. Proceedings of the 2012 Proceedings IEEE INFOCOM.

[16] Sinha S.K., Rangaraj N., Hemachandra N. (2010). Pricing surplus server capacity for mean waiting time sensitive customers. Eur. J. Oper. Res..

[17] Puterman M.L. (2005). Markov Decision Processes.

[18] Gouvy N., Hamouda E., Mitton N., Zorbas D. Energy efficient multi-flow routing in mobile Sensor Networks. Proceedings of the 2013 IEEE Wireless Communications and Networking Conference (WCNC).

[19] Zhang D., Li G., Zheng K., Ming X., Pan Z. (2014). An Energy-Balanced Routing Method Based on Forward-Aware Factor for Wireless Sensor Networks. IEEE Trans. Ind. Inform..

[20] Zhang D.G., Liu S., Zhang T., Liang Z. (2017). Novel unequal clustering routing protocol considering energy balancing based on network partition and distance for mobile education. J. Netw. Comput. Appl..

[21] Zhang D., Zheng K., Zhang T., Wang X. (2014). A novel multicast routing method with minimum transmission for WSN of cloud computing service. Soft Comput..

[22] Dressler F. (2007). Self-Organization in Sensor and Actor Networks.

[23] Pathan A. (2011). Security of Self-Organizing Networks: MANET, WSN, WMN, VANET.

[24] Gu X., Yu J., Yu D., Wang G., Lv Y. (2014). ECDC: An energy and coverage-aware distributed clustering protocol for wireless sensor networks. Comput. Electr. Eng..

[25] Le N.T., Jang M. (2015). Energy-efficient coverage guarantees scheduling and routing strategy for wireless sensor networks. Int. J. Distrib. Sens. Netw..

[26] More A., Raisinghani V. (2017). A survey on energy efficient coverage protocols in wireless sensor networks. J. King Saud Univ. Comput. Inf. Sci..

[27] Alghamdi T. (2020). Energy efficient protocol in wireless sensor network: Optimized cluster head selection model. Telecommun. Syst..

[28] Wang Z., Chen Y., Liu B. (2019). A sensor node scheduling algorithm for heterogeneous wireless sensor networks. Int. J. Distrib. Sens. Netw..

[29] Shih E., Cho S., Lee F.S., Calhoun B.H., Chandrakasan A. (2004). Design Considerations for Energy-Efficient Radios in Wireless Microsensor Networks. J. VLSI Signal Process. Syst. Signal Image Video Technol..

[30] Santhi V., Natarajan A.M. (2011). Active Queue Management Algorithm for TCP Networks Congestion Control. Eur. J. Sci. Res..

[31] Comer D.E. (2006). Internetworking with TCP/IP.

[32] Floyd S., Jacobson V. (1993). Random Early Detection (RED) gateways for Congestion Avoidance. IEEE/ACM Trans. Netw..

[33] Robertazzi T.G. (2012). Computer Networks and Systems: Queueing Theory and Performance Evaluation-Queueing Theorty and Performance Evaluation.

[34] Kleinrock L. (1976). Queueing Systems, Volume 2: Computer Applications.

[35] Kleinrock L. (1975). Queuing Systems, Volume 1: Theory.

[36] Lenin R.B., Ramaswamy S. (2015). Performance analysis of wireless sensor networks using queuing networks. Ann. Oper. Res..

[37] Adou Y., Markova E., Gudkova I. Performance Measures Analysis of Admission Control Scheme Model for Wireless Network, Described by a Queuing System Operating in Random Environment. Proceedings of the 2018 10th International Congress on Ultra Modern Telecommunications and Control Systems and Workshops (ICUMT).

[38] Borodakiy V.Y., Samouylov K.E., Gudkova I.A., Ostrikova D.Y., Ponomarenko-Timofeev A.A., Turlikov A.M., Andreev S.D. Modeling unreliable LSA operation in 3GPP LTE cellular networks. Proceedings of the 2014 6th International Congress on Ultra Modern Telecommunications and Control Systems and Workshops (ICUMT).

[39] Kempa W.M. (2019). Analytical Model of a Wireless Sensor Network (WSN) Node Operation with a Modified Threshold-Type Energy Saving Mechanism. Sensors.

[40] Ghosh S., Unnikrishnan S. Reduced power consumption in wireless sensor networks using queue based approach. Proceedings of the 2017 International Conference on Advances in Computing, Communication and Control (ICAC3).

[41] Moy J. Open Shortest Path First (OSPF). https://tools.ietf.org/html/rfc2328.

[42] Hedrick C. (1988). Routing Information Protocol (RIP).

[43] Zhang X., Zhou Z., Cheng D. (2017). Efficient path routing strategy for flows with multiple priorities on scale-free networks. PLoS ONE.

[44] Groenevelt R., Koole G., Nain P. (2002). On the bias vector of a two-class preemptive priority queue. Math. Methods Oper. Res..

[45] Brouns G.A., van der Wal J. (2006). Optimal threshold policies in a two-class preemptive priority queue with admission and termination control. Queueing Syst..

[46] Xu S. (1994). A duality approach to admission and scheduling controls of queues. Queueing Syst. Theory Appl..

[47] Righter R. (2000). Expulsion and scheduling control for multiclass queues with heterogeneous servers. Queueing Syst. Theory Appl..

[48] Brouns G., van der Wal J. (2003). Optimal threshold policies in a workload model with a variable number of service phases per job. Math. Methods Oper. Res..

[49] Puterman M. (1994). Markov Decision Processes: Discrete Stochastic Dynamic Programming.

[50] Lippman A.L. (1975). Applying a new device in the optimization of exponential queueing systems. Oper. Res..

[51] Khorov E., Kiryanov A., Loginov V., Lyakhov A. Head-of-line blocking avoidance in multimedia streaming over wireless networks. Proceedings of the 2014 IEEE 25th Annual International Symposium on Personal, Indoor, and Mobile Radio Communication (PIMRC).

[52] Elhafsi E.H., Molle M., Manjunath D. (2008). On the application of forking nodes to product-form queueing networks. Int. J. Commun. Syst..

[53] Kumar A., Manjunath D.K.J. (2004). Communication Networking: An Analytical Approach.

[54] Bellman R. (1957). Dynamic Programming.

[55] Howard R. (1960). Dynamic Programming and Markov Processes.

[56] Liu Z., Nain P., Towsley D. (1995). Sample path methods in the control of queues. Queueing Syst..

[57] Weber R.R., Stidham S. (1987). Optimal control of service rates in networks of queues. Adv. Appl. Probab..

[58] Bertsekas D.P. (2001). Dynamic Programming and Optimal Control.

[59] ElHafsi M., Fang J., Hamouda E. (2020). Optimal production and inventory control of multi-class mixed backorder and lost sales demand class models. Eur. J. Oper. Res..

